# Big Data-Based Identification of Multi-Gene Prognostic Signatures in Liver Cancer

**DOI:** 10.3389/fonc.2020.00847

**Published:** 2020-05-28

**Authors:** Meiliang Liu, Xia Liu, Shun Liu, Feifei Xiao, Erna Guo, Xiaoling Qin, Liuyu Wu, Qiuli Liang, Zerui Liang, Kehua Li, Di Zhang, Yu Yang, Xingxi Luo, Lei Lei, Jennifer Hui Juan Tan, Fuqiang Yin, Xiaoyun Zeng

**Affiliations:** ^1^School of Public Health, Guangxi Medical University, Nanning, China; ^2^Key Laboratory of Longevity and Ageing-Related Disease of Chinese Ministry of Education, Centre for Translational Medicine and School of Preclinical Medicine, Guangxi Medical University, Nanning, China; ^3^Department of Epidemiology and Biostatistics, University of South Carolina, Columbia, SC, United States; ^4^School of International Education, Guangxi Medical University, Nanning, China; ^5^School of Life Sciences and Chemical Technology, Ngee Ann Polytechnic, Singapore, Singapore; ^6^Life Sciences Institute, Guangxi Medical University, Nanning, China; ^7^Key Laboratory of High-Incidence-Tumor Prevention and Treatment, Guangxi Medical University, Ministry of Education, Nanning, China

**Keywords:** liver cancer, gene combinations, data mining, disease-free survival (DFS), overall survival (OS)

## Abstract

Simultaneous identification of multiple single genes and multi-gene prognostic signatures with higher efficacy in liver cancer has rarely been reported. Here, 1,173 genes potentially related to the liver cancer prognosis were mined with Coremine, and the gene expression and survival data in 370 samples for overall survival (OS) and 319 samples for disease-free survival (DFS) were retrieved from The Cancer Genome Atlas. Numerous survival analyses results revealed that 39 genes and 28 genes significantly associated with DFS and OS in liver cancer, including 18 and 12 novel genes that have not been systematically reported in relation to the liver cancer prognosis, respectively. Next, totally 9,139 three-gene combinations (including 816 constructed by 18 novel genes) for predicting DFS and 3,276 three-gene combinations (including 220 constructed by 12 novel genes) for predicting OS were constructed based on the above genes, and the top 15 of these four parts three-gene combinations were selected and shown. Moreover, a huge difference between high and low expression group of these three-gene combination was detected, with median survival difference of DFS up to 65.01 months, and of OS up to 83.57 months. The high or low expression group of these three-gene combinations can predict the longest prognosis of DFS and OS is 71.91 months and 102.66 months, and the shortest is 6.24 months and 13.96 months. Quantitative real-time polymerase chain reaction and immunohistochemistry reconfirmed that three genes *F2, GOT2*, and *TRPV1* contained in one of the above combinations, are significantly dysregulated in liver cancer tissues, low expression of *F2, GOT2*, and *TRPV1* is associated with poor prognosis in liver cancer. Overall, we discovered a few novel single genes and multi-gene combinations biomarkers that are closely related to the long-term prognosis of liver cancer, and they can be potential therapeutic targets for liver cancer.

## Introduction

Liver cancer is the sixth most common cancer and the fourth leading cause of cancer-related deaths (1). Specifically, hepatocellular carcinoma (HCC) accounts for more than 90% of liver cancer cases from a histopathological perspective. According to the GLOBOCAN 2018 database, there are about 841,000 new HCC cases and 782,000 related deaths worldwide each year, with China accounting for nearly half of the total number of global HCC cases and deaths (2, 3). In China, the Guangxi province has higher morbidity and mortality rates than the national average (4). The high mortality and poor prognosis of HCC poses a global challenge. Despite the slight increase in the 5-year survival rate of liver cancer in China from 10.1 to 12.1% over the periods of 2003–2015, it still remains at a low level (5). A survival analysis of 2, 887 liver cancer patients in 14 years showed that the 1-year, 3-year, and 5-year survival rates were 49.3, 26.6, and 19.5%, respectively (6).

Although there are many existing therapies for HCC including surgical resection, transplantation, ablation, and transcatheter chemoembolization, etc., the long-term survival of HCC patients remains poor due to their limited indications and different effects on prognosis (7–10). A 20-year prospective cohort analysis reported that the 5-year survival rates of TNM stage I, II, IIIA, and IVA patients after hepatectomy were 81.7, 77.2, 44, and 28.2%, respectively (11). Therefore, it is of crucial importance to explore new prognostic biomarkers and investigate treatment strategies to improve the overall prognosis of HCC patients.

Currently, the research on prognostic molecular markers of HCC is still ongoing, and many single-gene or multi-gene combination molecular markers related to HCC invasion, metastasis and prognosis are being gradually discovered. For example, the expression of *HMGA1* in HCC is associated with poor prognosis and is found to promote tumor growth and migration *in vitro* (12). The overexpression of *SYPL1* is associated with epithelial-mesenchymal transition (EMT) of HCC cells and can predict the prognosis of HCC (13). *RBM8A* and *SIRT5* promote the migration and invasion of HCC cells by activating the EMT signaling pathway and targeting *E2F1* (14, 15), respectively (16, 17). The *EpCAM* (18), a liver X receptor (*LXR*) (19), *SPAG5* (20), and *KOR* (21) have been shown to be strongly correlated with HCC metastasis, invasion, or prognosis. Arginase-1, *FTCD*, and *MOC-31* have a good performance in the diagnosis of HCC (22). *TMEM88, CCL14*, and *CLEC3B* can serve as potential prognostic markers of HCC (23). At the same time, some multi-gene combined prognostic studies on HCC have also been reported. For example, three genes (*UPB1, SOCS2, RTN3*) combination markers (24) and four genes (*CENPA, SPP1, MAGEB6, HOXD9*) combination models can predict the overall survival in patients with HCC prognosis (25).

However, due to the sample size limitation and the heterogeneity of the samples in different studies, the efficiency of the identified prognostic markers for liver cancer still has ample space to improve. In addition, because of the myriad of gene interaction capabilities and the possibility of synergistic promotion of disease progression, it is of great significance to find some multi-gene combinations that may have better prognostic efficacy than single genes for prognostic targets of liver cancer. Therefore, the leverage of the large sample sizes of the public data platforms, integrating new and effective mining and screening methods, as well as reliable experimental verification is a very promising direction for the discovery of multiple effective single genes and multi-gene combination prognostic markers of liver cancer.

High-throughput profiling technologies and bioinformatics methods are now being applied to all fields of biomedical research. A mass of cancer data, such as the mRNA expression, copy number variation, single nucleotide polymorphism (SNP), and microRNA expression generated by those tools are collected in public archives such as The Cancer Genome Atlas (TCGA) (http://cancergenome.nih.gov/), Coremine (http://www.coremine.com/medical/), Oncomine (https://www.oncomine.org/resource/login.html), Gene Expression Omnibus database (GEO, https://www.ncbi.nlm.nih.gov/geo/), etc. Making full use of the public data from these databases is meaningful for exploring and discovering effective HCC prognostic biomarkers. For instance, Li et al. (24) developed a three-gene prognostic signature composing of three genes *UPB1, SOCS2*, and *RTN3*, which was revealed to have prognostic value for HCC patients based on TCGA data. Our previous study used data retrieved from the Coremine, TCGA, and GEO database and discovered that high-expressed E2F transcription factor 3 is associated with poor prognosis of HCC (26).

In this study, we used text mining approach to find the medial related candidate gene list for liver cancer prognosis, and a total of 1,173 genes that might be related to the prognosis of liver cancer were finally obtained. The association of the 1,173 genes with overall survival (OS) and disease-free survival (DFS) was accessed in a large sample of TCGA cohort, in which the subgroups of 319 patients with DFS and 370 with OS were available. The survival analyses are carried out for each of these genes to identify single prognostic markers. Moreover, we performed survival analyses of the gene combinations and performed multiple screening for these HCC prognostic molecular markers, revealing the association between the expression of numerous genes or gene combinations and the survival in HCC patients. We then compared the ability of single genes and multiple gene combinations to predict the prognosis of HCC. Moreover, a huge difference between high and low expression group of these three-gene combinations was detected, with median survival difference of DFS up to 65.01 months, and of OS up to 83.57 months. The high or low expression group of these three-gene combinations can predict the longest prognosis of DFS and OS is 71.91 months and 102.66 months, and the shortest is 6.24 months and 13.96 months. Among the above genes that may be strongly correlated with the prognosis of HCC identified in large sample data, it was found that the combination of the three genes *F2, GOT2*, and *TRPV1* that have not been systematically reported has a strong ability to predict the prognosis of HCC. We further verified *F2, GOT2*, and *TRPV1* by three independent expression profile microarray data for liver cancer acquired from the Oncomine database, and conducted the quantitative real-time polymerase chain reaction (qRT-PCR) in 20 pairs of HCC and adjacent tissues, and immunohistochemistry (IHC) staining in 90 pairs of HCC and its precancerous tissues. These results validated that the low expression of *F2, GOT2*, and *TRPV1* in liver cancer was associated with the poor prognosis of liver cancer.

## Materials and Methods

### Data Sources

We combined 3 corresponding concepts of the key word “liver cancer” with 2 concepts of the key word “prognosis” and 10 concepts of the key word “outcome,” respectively, (Supplementary Table S1), and searched for their corresponding genes or proteins in the Coremine database (http://www.coremine.com/medical/). After deleting duplicates, we selected 1,173 gene entries with *p*-values < 0.05 that might be associated with the prognosis of liver cancer for further analyses (Supplementary Table S2).

The above genes mined in the Coremine database include some genes obtained from other gene-mining reports; however, the number of samples and data standards in each report is different. Therefore, we selected the cohort of The Cancer Genome Atlas (TCGA) (http://cancergenome.nih.gov/), a database with consistent sample size and data standards, to conduct unified batch verification of these genes and conduct three-gene combinations survival analyses.

We studied the relationship between each of the selected 1,173 genes and the prognosis of liver cancer patients in TCGA cohort which downloaded from cBioPortal for Cancer Genomics (https://www.cbioportal.org/) in September 2018 (27, 28), and a subgroup of 319 liver cancer samples with HCC DFS corresponding follow-up data and a subgroup of 370 liver cancer samples with HCC OS corresponding follow-up data were chosen.

### Survival Analysis and Gene Selection

Kaplan-Meier estimation of survival functions and Log-rank tests were used to evaluate effect of genes on DFS and OS. The Cox proportional hazard model was performed for multivariate analyses of HCC prognosis. Survival analyses were performed using the R survival package in R (version 3.3.1). The Kaplan-Meier survival curves and Cox proportional hazards regression model for DFS and OS were generated by IBM SPSS (version 23.0). The median expression level of a gene was used as a cutoff value for the classification of patients into high and low expression groups (29).

### Human Tissue Samples

For the validation studies, we used 20 patients who underwent primary and curative hepatectomy from Apr 2016 to Apr 2018 at the First Affiliated Hospital of Guangxi Medical University. Those patients who have distinctive pathologic diagnosis of HCC without preoperative anticancer treatment were eligible for inclusion in this study. The paraffin-embedded pathologic specimens were collected during surgery and stored in a liquid nitrogen tank until the step of mRNA isolation. All patients received an explanation for the purpose of the study and signed informed consent. The Ethics Committee of Guangxi Medical University granted approval for this study. For IHC, a commercial biological tissue microarray containing 90 pairs of HCC and adjacent normal liver tissues was constructed by the Biological sample library of Shanghai Outdo Biotech Company, and the survival information of each case was usable. (Microarray: HLivH180Su14).

### Quantitative Real-Time Polymerase Chain Reaction (qRT-PCR)

QRT-PCR was performed to evaluate the mRNA expression of selected genes in 20 HCC and their matched precancerous tissues. Total RNA was isolated using Trizol reagent (Life Technologies, Inc., NY, USA) according to the manufacturer's instructions. The concentration and purity of the total RNA were detected using Microplate reader (Bioteck Instruments, Inc., VT, USA). RNA reverse transcription was then performed with the PrimeScript™ RT reagent Kit (Takara Biomedical Technology (Beijing) Co., Ltd.) with gDNA Eraser (Perfect Real Time), and qRT-PCR was performed using the TB GreenTM Premix Ex TaqTM II (Tli RNaseH Plus) kit (Takara Biomedical Technology (Beijing) Co., Ltd.) protocol in a StepOnePlus system (Applied Biosystems. Life Technologies Holdings Pte Ltd, Singapore).

The sequences of the primers are as follows: *F2*: forward primer, 5′-CTGAGGGTCTGGGTACGAACT-3′, reverse primer, 5′-TGGGTAGCGACTCCTCCATAG-3′; *GOT2*: forward primer, 5′-AAGAGTGGCCGGTTTGTCAC-3′, reverse primer, 5′-AGAAAGACATCTCGGCTGAACT-3′; *TRPV1*: forward primer, 5′-TGCACGACGGACAGAACAC-3′, reverse primer, 5′-GCGTTGACAAGCTCCTTCAG-3′. The cycle conditions are as follows: after an initial incubation at 95°C for 30 s, the samples were cycled 40 times at 95°C for 5 s and 60°C for 30 s. The relative expression level of each gene in the individual samples was calculated using the 2^−^ΔΔ*Ct* method and normalized using GAPDH as an endogenous control.

### Immunohistochemistry (IHC)

EnVision™ FLEX+, Mouse, High pH, (Link) (K8002, Dako) was used for the immunohistochemistry. After the tissue chips were baked and placed in LEICAST5010 (LEICA), PT Link (Dako North America, Inc.) was used for antigen retrieval. Primary antibodies were diluted (*F2*, 1:3000; *GOT2*, 1:80000; *TRPV1*, 1:1500) and incubated overnight at 4°C. The secondary antibody reactions were carried out using the Autostainer Link 48 (Dako North America, Inc.), the sections were subjected to color development with the DAB chromogenic kit, and finally counterstained with Hematoxylin (SLBT4555, Sigma Aldrich). The following antibodies were used: *F2*, 1: Anti-Thrombin (ab83981; Abcam), *GOT2*, 1: Anti-FABP-1 (ab171739; Abcam), *TRPV1*, 1: Anti-VR1 (ab3487; Abcam). All slides were evaluated by two independent pathologists who were blind about the clinicopathologic data.

The expression levels were scored as the staining intensity (0, negative; 1+, weak; 2+, moderate; 3+, strong) multiplied by the proportion of immunopositive staining area (0, < 25%; 1+, 25–50%; 2+, 50–75%; 3+, >75%) intensity of staining. Expression scores <5 were considered as “low expression,” and scores ≥5 were considered as “high expression.”

### Statistics

Statistical analyses were conducted using R 3.3.1 (Auckland, NZ) and IBM SPSS 23.0 (Chicago, USA). McNemar test was used to test the paired 4-fold table experimental data of IHC. The paired *t*-test was used to analyze the qRT-PCR experimental data. Except for single-gene survival analyses and three-gene prognosis survival analyses with *p*-value < 0.01 as statistically significant, other statistical analyses were considered statistically significant with two-sided *p*-value < 0.05.

## Results

### Selection of Genes Related to Liver Cancer Prognosis and Liver Cancer Samples

We combined 3 corresponding concepts of the key word “liver cancer” [Liver neoplasms (alias Liver Cancer) (disease) (60,666 connections); Liver carcinoma (alias liver cell cancer) (disease) (55,739 connections); Carcinoma, Hepatocellular (alias Adult Liver Cancer) (mesh) (57,034 connections)] with 2 corresponding concepts of the key word “prognosis” [Prognosis (mesh) (77,312 connections); Prognostic Marker (alias Prognosis Marker) (chemical) (22,056 connections)] and 10 corresponding concepts of the key word “outcome” [Fatal Outcome (mesh) (34,016 connections); Outcome Assessment (Health Care) (alias Outcome Study) (mesh) (48,296 connections); Outcome studies (procedure) (9,545 connections); Treatment Outcome (mesh) (77,246 connections); Outcomes research (procedure) (5,540 connections); Outcome monitoring (procedure) (2,030 connections); Patient-focused outcomes (procedure) (3,830 connections); Treatment outcome in HSR (procedure) (998 connections); Patient Reported Outcome Measures (alias Patient Reported Outcome) (mesh) (2,301 connections); Patient Outcome Assessment (mesh) (9,066 connections)], respectively, (Supplementary Table S1), and searched for their corresponding genes or proteins in the Coremine database (http://www.coremine.com/medical/). With *p*-values < 0.05 as the criteria, a total of 1,173 genes that might be related to the prognosis of liver cancer were finally obtained after screening and elimination of duplicates. As the samples of liver cancer in the Coremine database were not uniform enough, we selected 319 samples for DFS and 370 samples for OS of liver cancer from the TCGA database and obtained the corresponding survival data as well as the expression information of the above 1,173 genes in these samples. This was necessary to carry out the subsequent survival analyses of these genes for liver cancer.

### The Single Genes Prognostic Analyses

**Figure 1:**
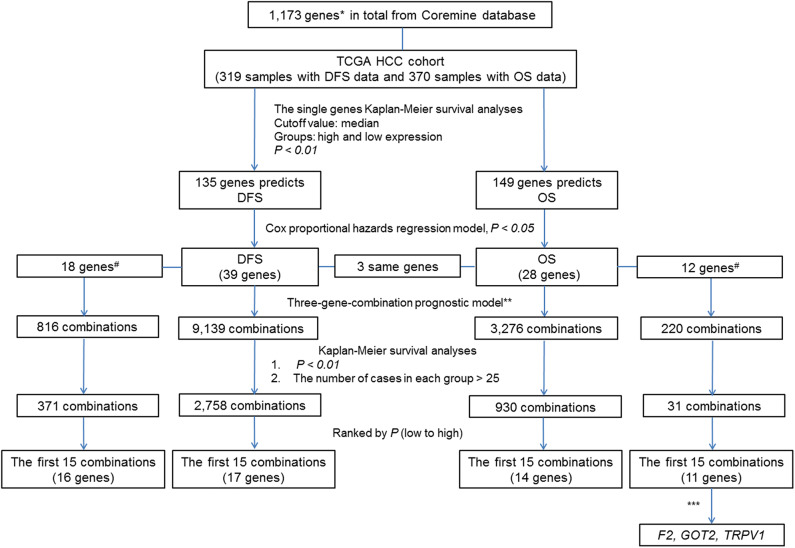
Flow Chart. ^#^The associations of the genes with liver cancer prognosis that were not previously reported. *By text mining of searching for key words related to the markers of liver cancer prognosis and screening, a total of 1,173 genes containing both previously reported and unreported associations with liver cancer prognosis were obtained. **In each sample, the simultaneous high expression of all three genes was considered as high expression group in new combination. Similarly, the simultaneous low expression of all three genes was considered as low expression group in new combination. ***By comparing the prognostic value of individual genes and their combinations, we selected genes of combination *F2- GOT2-TRPV1* for subsequent verification.

To clearly describe our process of screening genes, a flowchart of the analysis procedure was developed (Figure 1). First, we performed the Kaplan-Meier analysis of each of the 1,173 genes. It was found that the mRNA expression of 276 genes and 283 genes was significantly associated with DFS in 319 patients (*p* < 0.05) and OS in 370 patients (*p* < 0.05), respectively. Additionally, the mRNA expression of 166 of these genes was significantly associated with both DFS and OS (*p* < 0.05).

To further investigate the value of the genes in the prognosis of liver cancer, we chose 135 genes and 149 genes with *p*-values < 0.01 for DFS and OS, respectively. Next, we used the Cox proportional hazards regression model to employ multivariate analyses on the above genes, respectively to determine the DFS and OS prediction potential of these genes.

The DFS-related multivariate analysis results showed that the expression of 39 genes (*ALDOB, APOB, AURKB, C5, CCNF, CD4, CENPJ, CETP, COL18A1, CPT2, DAND5, DNASE1, EBPL, F7, FLT3, G6PD, GNMT, ITGB2, KLRK1, KNG1, LMOD1, NEK2, PCLAF, PER1, PKM, POU2F1, PPAT, PPIA, PRF1, PTPN6, RUNX3, SELP, SLCO1B1, SPPL2A, STAT5A, TCF21, TRPV1, TUSC1*, and *TYMS*) was significantly associated with DFS in HCC patients (*p* < 0.05, Table 1). The highly significant results of both the DFS-related single-gene survival analyses for each of these 39 genes and multivariate analysis confirmed that the above 39 genes have a strong association with the DFS of liver cancer, especially the 5-year disease free survival rate of liver cancer.

**Table 1:** Multivariate analyses of prognosis of DFS of 319 HCC patients and OS of 370 HCC patients in a TCGA cohort.

**Items**	**Genes**	**B**	**SE**	**Wald**	**Sig**.	**Exp (B)**	**95.0% CI**	
							**Lower**	**Upper**
DFS associated	*ALDOB*	−0.580	0.186	9.750	0.002	0.560	0.389	0.806
	*APOB*	−0.436	0.217	4.023	0.045	0.647	0.423	0.990
	*AURKB*	0.527	0.211	6.208	0.013	1.694	1.119	2.564
	C5^*^	−0.420	0.170	6.093	0.014	0.657	0.471	0.917
	*CCNF*	0.694	0.334	4.310	0.038	2.002	1.040	3.857
	CD4^*^	−0.774	0.316	6.007	0.014	0.461	0.248	0.856
	*CENPJ*	1.053	0.243	18.794	0.000	2.867	1.781	4.615
	CETP^*^	0.829	0.423	3.851	0.050	2.291	1.001	5.245
	COL18A1^*^	0.417	0.207	4.064	0.044	1.518	1.012	2.278
	*CPT2*	0.558	0.247	5.114	0.024	1.747	1.077	2.834
	DAND5^*^	−0.427	0.183	5.466	0.019	0.652	0.456	0.933
	DNASE1^*^	0.382	0.136	7.927	0.005	1.465	1.123	1.910
	EBPL^*^	−0.766	0.280	7.463	0.006	0.465	0.268	0.805
	F7^*^	−0.496	0.175	8.034	0.005	0.609	0.432	0.858
	FLT3^*^	−0.700	0.240	8.512	0.004	0.497	0.310	0.795
	*G6PD*	0.477	0.188	6.438	0.011	1.611	1.115	2.328
	*GNMT*	0.427	0.160	7.118	0.008	1.533	1.120	2.097
	ITGB2^*^	1.112	0.301	13.662	0.000	3.042	1.686	5.486
	*KLRK1*	0.932	0.384	5.883	0.015	2.539	1.196	5.390
	KNG1^*^	0.645	0.277	5.412	0.020	1.906	1.107	3.282
	LMOD1^*^	−0.873	0.410	4.524	0.033	0.418	0.187	0.934
	*NEK2*	−0.546	0.263	4.299	0.038	0.579	0.346	0.971
	*PCLAF*	0.526	0.243	4.700	0.030	1.693	1.052	2.724
	*PER1*	−0.670	0.221	9.169	0.002	0.512	0.332	0.790
	*PKM*	−0.645	0.282	5.210	0.022	0.525	0.302	0.913
	*POU2F1*	0.455	0.142	10.236	0.001	1.577	1.193	2.084
	PPAT^*^	0.966	0.210	21.121	0.000	2.628	1.741	3.969
	PPIA^*^	0.626	0.183	11.661	0.001	1.870	1.306	2.679
	PRF1^*^	−1.676	0.370	20.505	0.000	0.187	0.091	0.386
	*PTPN6*	−0.610	0.227	7.203	0.007	0.543	0.348	0.848
	*RUNX3*	0.967	0.375	6.659	0.010	2.629	1.262	5.479
	SELP^*^	0.790	0.270	8.587	0.003	2.203	1.299	3.736
	*SLCO1B1*	−0.524	0.213	6.029	0.014	0.592	0.390	0.900
	SPPL2A^*^	−0.669	0.217	9.528	0.002	0.512	0.335	0.783
	*STAT5A*	−1.704	0.489	12.149	0.000	0.182	0.070	0.474
	*TCF21*	−0.979	0.401	5.961	0.015	0.376	0.171	0.824
	TRPV1^*^	−0.520	0.189	7.604	0.006	0.595	0.411	0.860
	*TUSC1*	0.423	0.188	5.044	0.025	1.526	1.055	2.207
	*TYMS*	0.523	0.245	4.558	0.033	1.687	1.044	2.727
OS associated	*ABCC1*	1.097	0.369	8.841	0.003	2.994	1.453	6.168
	ANXA7^*^	−0.554	0.201	7.618	0.006	0.575	0.388	0.852
	*APOB*	−0.791	0.311	6.461	0.011	0.453	0.246	0.834
	*ATG7*	0.613	0.312	3.876	0.049	1.847	1.003	3.400
	*BAK1*	−0.490	0.231	4.497	0.034	0.613	0.390	0.964
	*CA9*	0.761	0.363	4.399	0.036	2.140	1.051	4.356
	*CCNA2*	0.502	0.203	6.094	0.014	1.652	1.109	2.461
	*CHD1L*	0.491	0.181	7.377	0.007	1.634	1.147	2.330
	*CYP3A4*	0.999	0.364	7.539	0.006	2.717	1.331	5.544
	*E2F1*	0.360	0.172	4.371	0.037	1.433	1.023	2.008
	*EZH2*	0.985	0.399	6.103	0.013	2.678	1.226	5.852
	F2^*^	0.711	0.313	5.174	0.023	2.036	1.103	3.757
	*G6PC*	−0.677	0.341	3.937	0.047	0.508	0.260	0.992
	*GMPS*	0.733	0.291	6.345	0.012	2.081	1.177	3.681
	GOT2^*^	−1.509	0.484	9.723	0.002	0.221	0.086	0.571
	*HDAC2*	0.813	0.316	6.628	0.010	2.255	1.214	4.187
	HPX^*^	0.930	0.384	5.882	0.015	2.535	1.195	5.378
	*KPNA2*	0.835	0.284	8.664	0.003	2.305	1.322	4.018
	*LAPTM4B*	−0.492	0.168	8.616	0.003	0.611	0.440	0.849
	MAGEB3^*^	0.393	0.179	4.824	0.028	1.482	1.043	2.105
	MAPT^*^	0.660	0.243	7.349	0.007	1.934	1.201	3.117
	MPV17^*^	1.141	0.488	5.468	0.019	3.129	1.203	8.141
	NTF3^*^	1.089	0.357	9.318	0.002	2.973	1.477	5.983
	PPAT^*^	0.752	0.286	6.897	0.009	2.122	1.210	3.719
	SLC2A1^*^	−0.921	0.440	4.383	0.036	0.398	0.168	0.943
	SLC38A1^*^	−0.768	0.289	7.063	0.008	0.464	0.263	0.817
	*SPP1*	0.604	0.264	5.219	0.022	1.830	1.090	3.073
	TRPV1^*^	0.453	0.201	5.044	0.025	1.572	1.059	2.334

* *The gene has not been systematically reported to be associated with HCC prognosis*.

*Cox proportional hazard model was used to analyze the impact of 135 genes on DFS and the impact of 149 genes on OS, respectively, P < 0.05 were considered to be significant*.

*39 genes and 28 genes were significantly associated with liver cancer DFS and OS, respectively*.

The OS-related multivariate analysis results showed that the expression of 28 genes (*ABCC1, ANXA7, APOB, ATG7, BAK1, CA9, CCNA2, CHD1L, CYP3A4, E2F1, EZH2, F2, G6PC, GMPS, GOT2, HDAC2, HPX, KPNA2, LAPTM4B, MAGEB3, MAPT, MPV17, NTF3, PPAT, SLC2A1, SLC38A1, SPP1*, and *TRPV1*) was significantly associated with OS in HCC patients. (*p* < 0.05, Table 1). The strongly significant results of both the OS-related single-gene survival analyses and multivariate analysis confirmed that these 28 genes are significantly associated with the OS of liver cancer, especially the 5-year survival rate of liver cancer.

Additionally, among the above-mentioned genes selected after single-gene survival analyses and multivariate analyses, 3 genes (*APOB, PPAT*, and *TRPV1*) were significantly associated with both DFS and OS in HCC patients.

Heat maps of the expression of the above 39 DFS-related genes and 28 OS-related genes in 1173 TCGA liver cancer samples, respectively, which grouped by prognosis status, were shown in Supplementary Figure S1.

### Three-Gene-Combination Prognostic Model

To reflect the association of the expression of the combined genes with the prognosis of HCC, three-gene-combinations of the above 39 and 28 single genes that are significantly associated with DFS and OS, respectively, were formed, resulting in 9,139 and 3,276 three-gene-combinations for DFS and OS, respectively. In each combination, simultaneous high expression of the three genes in the same case was defined as the co-high expression group. Similarly, simultaneous low expression of the three genes in the same case was considered to be the co-low expression group. In order to ensure the comparability between the high and the low expression group, we deleted combinations which had < 25 cases in the co-high or co-low expression group.

#### Three-Gene-Combination of Prediction for DFS in Liver Cancer

K-M survival analysis of each of the above 9,139 combinations constituted by 39 DFS-related single genes was first performed. Then, we selected a total of 2,758 combinations with *p*-values < 0.01, excluding the combinations with no more than 25 cases in the co-high expression or co-low expression groups. Apparently, these selected 2,758 combinations have significant prognostic implications for DFS in liver cancer.

In addition, 18 of the above 39 single genes have not yet been systematically reported to be associated with HCC prognosis, and these 18 genes can combine into 816 three-gene-combinations. The results of the K-M survival analyses showed that 317 combinations had significant association with DFS of liver cancer (*p* < 0.01).

**Figure 2:**
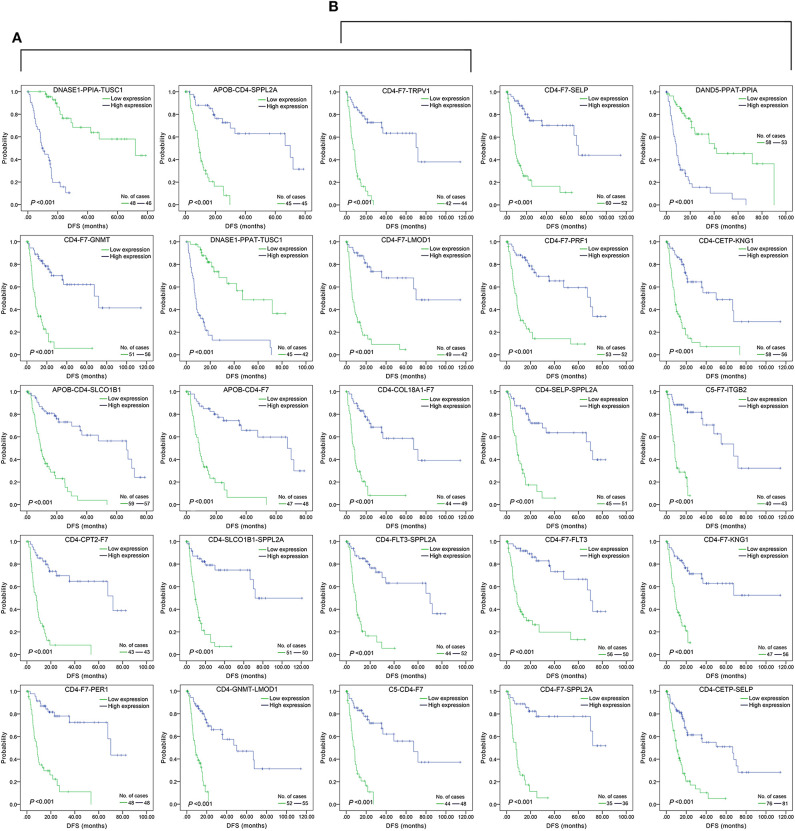
Association of DFS and the top 15 three-gene-combinations with smallest *p-*values, using the data of HCC samples in a TCGA cohort and assessed by Kaplan-Meier analyses. The high expression group (blue line) of the combination consisted of samples with high expression of all three genes, and the low expression group (green line) of the combination consisted of samples with low expression of all three genes. The number of high and low expression groups in each combination was >25. **(A)** Association of DFS and the top 15 combinations of the overall genes combinations. **(B)** Association of DFS and the top 15 combinations of the unreported genes combinations.

**Figure 3:**
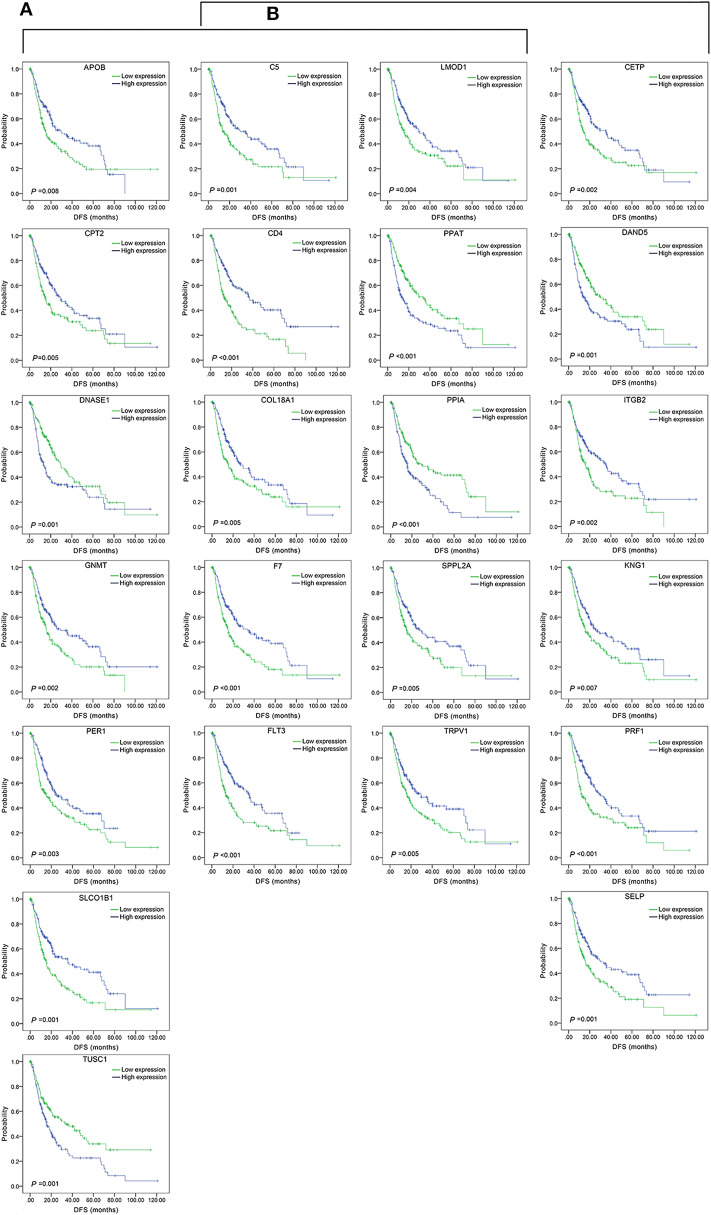
Association of DFS and the individual genes contained in the top 15 combinations with the lowest *P*-values, using the data of HCC samples in a TCGA cohort and assessed by Kaplan-Meier analyses. **(A)** Association of DFS and the 17 single genes contained in the first 15 total-gene combinations. **(B)** Association of DFS and the 16 single genes contained in the first 15 unreported-gene combinations.

The top 15 combinations of the above 2,758 and 317 combinations with the smallest *p*-values were chosen. The DFS-related survival analyses diagrams and tables of these combinations and the single genes they contain are as follows (Figures 2, 3; Tables 2, 3).

**Table 2:** The associations of three-gene combinations with disease-free survival (DFS) of HCC patients in a TCGA cohort, analyzed by Kaplan-Meier method.

**DFS (Median) of combinations of 39 genes with HCC prognosis**									**DFS (Median) of combinations of 18 genes have unknown association with HCC prognosis**							
		**Estimate**	**Std. Error**	**95% confidence interval**		***P***	**Median survival time difference (H-L)**				**Estimate**	**Std. Error**	**95% confidence interval**		***P***	**Median survival time difference (H-L)**
				**Lower boundary**	**Upper boundary**								**Lower boundary**	**Upper boundary**		
*DNASE1-PPIA-TUSC1*	H	9.490	1.597	6.360	12.620	0.000	−62.420		*CD4-F7-TRPV1*	H	71.910	20.619	31.498	112.322	0.000	65.010
	L	71.910	24.365	24.154	119.666					L	6.900	1.657	3.652	10.148		
	Overall	21.620	4.848	12.119	31.121					Overall	15.740	5.309	5.334	26.146		
*CD4-F7-TRPV1*	H	71.910	20.619	31.498	112.322	0.000	65.010		*CD4-F7-LMOD1*	H	70.070	–	–	–	0.000	63.830
	L	6.900	1.657	3.652	10.148					L	6.240	1.408	3.480	9.000		
	Overall	15.740	5.309	5.334	26.146					Overall	17.640	3.833	10.127	25.153		
*CD4-F7-GNMT*	H	71.910	22.303	28.196	115.624	0.000	63.370		*CD4-COL18A1-F7*	H	67.580	21.110	26.205	108.955	0.000	59.660
	L	8.540	1.241	6.108	10.972					L	7.920	1.658	4.670	11.170		
	Overall	21.160	4.039	13.244	29.076					Overall	19.190	3.616	12.104	26.276		
*CD4-F7-LMOD1*	H	70.070	–	–	–	0.000	63.830		*CD4-FLT3-SPPL2A*	H	70.070	18.005	34.779	105.361	0.000	62.220
	L	6.240	1.408	3.480	9.000					L	7.850	1.486	4.937	10.763		
	Overall	17.640	3.833	10.127	25.153					Overall	19.650	7.275	5.391	33.909		
*CD4-COL18A1-F7*	H	67.580	21.110	26.205	108.955	0.000	59.660		*C5-CD4-F7*	H	67.580	15.374	37.447	97.713	0.000	59.660
	L	7.920	1.658	4.670	11.170					L	7.920	1.414	5.149	10.691		
	Overall	19.190	3.616	12.104	26.276					Overall	21.160	5.704	9.981	32.339		
*APOB-CD4-SLCO1B1*	H	66.620	13.239	40.672	92.568	0.000	57.130		*CD4-F7-SELP*	H	71.910	3.184	65.669	78.151	0.000	63.200
	L	9.490	0.918	7.691	11.289					L	8.710	0.783	7.176	10.244		
	Overall	19.650	4.976	9.897	29.403					Overall	21.550	8.496	4.898	38.202		
*CD4-CPT2-F7*	H	71.910	21.206	30.347	113.473	0.000	64.060		*CD4-F7-PRF1*	H	70.070	15.899	38.908	101.232	0.000	61.500
	L	7.850	2.024	3.883	11.817					L	8.570	1.055	6.502	10.638		
	Overall	15.700	2.776	10.259	21.141					Overall	21.160	3.455	14.389	27.931		
*CD4-F7-PER1*	H	70.070	2.855	64.475	75.665	0.000	61.500		*CD4-SELP-SPPL2A*	H	70.070	3.849	62.525	77.615	0.000	61.500
	L	8.570	1.225	6.170	10.970					L	8.570	0.819	6.964	10.176		
	Overall	25.300	8.227	9.175	41.425					Overall	18.590	5.837	7.149	30.031		
*APOB-CD4-SPPL2A*	H	70.070	23.928	23.171	116.969	0.000	60.940		*CD4-F7-FLT3*	H	70.070	3.048	64.097	76.043	0.000	61.360
	L	9.130	0.855	7.455	10.805					L	8.710	1.311	6.140	11.280		
	Overall	19.190	4.689	10.000	28.380					Overall	35.580	12.142	11.781	59.379		
*CD4-FLT3-SPPL2A*	H	70.070	18.005	34.779	105.361	0.000	62.220		*CD4-F7-SPPL2A*	H	–	–	–	–	0.000	–
	L	7.850	1.486	4.937	10.763					L	7.920	1.864	4.266	11.574		
	Overall	19.650	7.275	5.391	33.909					Overall	24.770	19.276	0.000	62.551		
*DNASE1-PPAT-TUSC1*	H	7.420	1.115	5.235	9.605	0.000	−39.620		*DAND5-PPAT-PPIA*	H	8.540	0.797	6.978	10.102	0.000	−33.480
	L	47.040	17.350	13.035	81.045					L	42.020	15.014	12.592	71.448		
	Overall	21.160	5.101	11.162	31.158					Overall	19.250	2.763	13.834	24.666		
*APOB-CD4-F7*	H	67.580	13.500	41.120	94.040	0.000	58.840		*CD4-CETP-KNG1*	H	50.030	14.498	21.614	78.446	0.000	41.550
	L	8.740	0.884	7.007	10.473					L	8.480	0.769	6.972	9.988		
	Overall	24.770	9.057	7.018	42.522					Overall	18.330	1.894	14.617	22.043		
*CD4-SLCO1B1-SPPL2A*	H	71.910	–	–	–	0.000	62.420		*C5-F7-ITGB2*	H	67.580	14.028	40.084	95.076	0.000	59.010
	L	9.490	1.171	7.194	11.786					L	8.570	1.316	5.991	11.149		
	Overall	19.650	6.519	6.873	32.427					Overall	35.580	9.185	17.577	53.583		
*C5-CD4-F7*	H	67.580	15.374	37.447	97.713	0.000	59.660		*CD4-F7-KNG1*	H	–	–	–	–	0.000	–
	L	7.920	1.414	5.149	10.691					L	8.740	1.206	6.376	11.104		
	Overall	21.160	5.704	9.981	32.339					Overall	21.550	8.293	5.295	37.805		
*CD4-GNMT-LMOD1*	H	50.030	16.348	17.987	82.073	0.000	41.550		*CD4-CETP-SELP*	H	66.620	14.883	37.450	95.790	0.000	56.370
	L	8.480	1.430	5.677	11.283					L	10.250	1.315	7.672	12.828		
	Overall	18.330	2.734	12.971	23.689					Overall	18.330	1.469	15.452	21.208		

**Table 3:** The associations of single genes contained in the multi-gene combinations with disease-free survival (DFS) and overall survival (OS) of HCC patients in a TCGA cohort, analyzed by Kaplan-Meier method.

**DFS (Median) of single genes of the combinations with HCC prognosis**									**OS (Median) of single genes of the combinations with HCC prognosis**							
		**Estimate**	**Std. Error**	**95% confidence interval**		***P***	**Median survival time difference (H-L)**				**Estimate**	**Std. Error**	**95% Confidence Interval**		***P***	**Median survival time difference (H-L)**
				**Lower boundary**	**Upper boundary**								**Lower boundary**	**Upper boundary**		
*APOB*	H	29.300	6.376	16.802	41.798	0.008	14.450		*ANXA7*	H	83.180	15.496	52.807	113.553	0.006	36.430
	L	14.850	2.049	10.834	18.866					L	46.750	7.280	32.481	61.019		
	Overall	20.930	2.318	16.387	25.473					Overall	55.650	7.925	40.116	71.184		
*C5*	H	29.960	6.762	16.706	43.214	0.001	16.330		*ATG7*	H	45.070	8.031	29.330	60.810	0.009	−35.610
	L	13.630	2.870	8.006	19.254					L	80.680	10.533	60.036	101.324		
	Overall	20.930	2.318	16.387	25.473					Overall	55.650	7.925	40.116	71.184		
*CD4*	H	36.700	7.693	21.622	51.778	0.000	23.070		*CA9*	H	37.290	8.317	20.989	53.591	0.000	−32.720
	L	13.630	2.089	9.536	17.724					L	70.010	10.210	49.999	90.021		
	Overall	20.930	2.318	16.387	25.473					Overall	55.650	7.925	40.116	71.184		
*CETP*	H	35.580	5.896	24.023	47.137	0.002	21.450		*CCNA2*	H	45.070	10.298	24.885	65.255	0.001	−24.940
	L	14.130	1.799	10.605	17.655					L	70.010	11.730	47.019	93.001		
	Overall	20.930	2.318	16.387	25.473					Overall	55.650	7.925	40.116	71.184		
*COL18A1*	H	27.200	4.885	17.625	36.775	0.005	11.600		*CHD1L*	H	39.750	6.940	26.148	53.352	0.006	−40.930
	L	15.600	3.114	9.497	21.703					L	80.680	6.587	67.770	93.590		
	Overall	20.930	2.318	16.387	25.473					Overall	55.650	7.925	40.116	71.184		
*CPT2*	H	29.300	4.767	19.956	38.644	0.005	14.350		*EZH2*	H	37.290	10.181	17.335	57.245	0.000	−43.390
	L	14.950	1.836	11.352	18.548					L	80.680	10.816	59.480	101.880		
	Overall	20.930	2.318	16.387	25.473					Overall	55.650	7.925	40.116	71.184		
*DAND5*	H	13.630	2.561	8.610	18.650	0.001	−16.330		*F2*	H	69.510	11.842	46.300	92.720	0.005	23.620
	L	29.960	5.455	19.269	40.651					L	45.890	7.020	32.132	59.648		
	Overall	20.930	2.318	16.387	25.473					Overall	55.650	7.925	40.116	71.184		
*DNASE1*	H	13.140	1.997	9.226	17.054	0.001	−16.160		*GMPS*	H	45.070	9.667	26.123	64.017	0.003	−24.440
	L	29.300	4.256	20.958	37.642					L	69.510	10.308	49.306	89.714		
	Overall	20.930	2.318	16.387	25.473					Overall	55.650	7.925	40.116	71.184		
*F7*	H	33.900	8.191	17.846	49.954	0.000	18.490		*GOT2*	H	70.010	12.025	46.441	93.579	0.000	32.260
	L	15.410	1.485	12.500	18.320					L	37.750	9.383	19.360	56.140		
	Overall	20.930	2.318	16.387	25.473					Overall	55.650	7.925	40.116	71.184		
*FLT3*	H	35.580	3.640	28.446	42.714	0.000	22.440		*HPX*	H	69.510	10.518	48.894	90.126	0.002	23.620
	L	13.140	1.833	9.547	16.733					L	45.890	10.112	26.070	65.710		
	Overall	20.930	2.318	16.387	25.473					Overall	55.650	7.925	40.116	71.184		
*GNMT*	H	29.300	9.167	11.334	47.266	0.002	13.370		*HDAC2*	H	45.070	8.365	28.675	61.465	0.002	−35.610
	L	15.930	1.821	12.360	19.500					L	80.680	12.796	55.599	105.761		
	Overall	20.930	2.318	16.387	25.473					Overall	55.650	7.925	40.116	71.184		
*ITGB2*	H	35.580	4.232	27.285	43.875	0.002	19.840		*KPNA2*	H	33.020	8.165	17.017	49.023	0.000	−47.660
	L	15.740	2.671	10.504	20.976					L	80.680	6.908	67.139	94.221		
	Overall	20.930	2.318	16.387	25.473					Overall	55.650	7.925	40.116	71.184		
*KNG1*	H	25.300	6.478	12.603	37.997	0.007	9.600		*LAPTM4B*	H	45.070	10.511	24.468	65.672	0.000	−35.610
	L	15.700	2.458	10.882	20.518					L	80.680	12.598	55.988	105.372		
	Overall	20.930	2.318	16.387	25.473					Overall	55.650	7.925	40.116	71.184		
*LMOD1*	H	29.660	5.120	19.625	39.695	0.004	13.960		*MAPT*	H	41.750	6.888	28.249	55.251	0.006	−28.260
	L	15.700	2.655	10.497	20.903					L	70.010	9.844	50.716	89.304		
	Overall	20.930	2.318	16.387	25.473					Overall	55.650	7.925	40.116	71.184		
*PER1*	H	25.490	6.529	12.694	38.286	0.003	10.080		*MPV17*	H	37.290	6.644	24.268	50.312	0.000	−43.390
	L	15.410	3.485	8.579	22.241					L	80.680	6.504	67.933	93.427		
	Overall	20.930	2.318	16.387	25.473					Overall	55.650	7.925	40.116	71.184		
*PPAT*	H	14.130	2.656	8.924	19.336	0.000	−19.770		*NTF3*	H	70.010	12.704	45.110	94.910	0.002	29.640
	L	33.900	5.401	23.314	44.486					L	40.370	8.143	24.409	56.331		
	Overall	20.930	2.318	16.387	25.473					Overall	55.650	7.925	40.116	71.184		
*PPIA*	H	15.600	1.475	12.709	18.491	0.000	−13.280		*PPAT*	H	58.840	14.928	29.580	88.100	0.009	−10.670
	L	28.880	7.575	14.033	43.727					L	69.510	11.354	47.256	91.764		
	Overall	20.930	2.318	16.387	25.473					Overall	55.650	7.925	40.116	71.184		
*PRF1*	H	29.960	4.358	21.418	38.502	0.000	17.350		*SLC2A1*	H	45.890	6.187	33.763	58.017	0.000	−37.290
	L	12.610	2.004	8.681	16.539					L	83.180	17.113	49.638	116.722		
	Overall	20.930	2.318	16.387	25.473					Overall	55.650	7.925	40.116	71.184		
*SELP*	H	29.960	6.294	17.624	42.296	0.001	14.260		*SLC38A1*	H	45.070	3.919	37.389	52.751	0.001	−35.610
	L	15.700	2.465	10.868	20.532					L	80.680	7.141	66.684	94.676		
	Overall	20.930	2.318	16.387	25.473					Overall	55.650	7.925	40.116	71.184		
*SLCO1B1*	H	35.840	10.368	15.518	56.162	0.000	20.890		*SPP1*	H	40.370	5.288	30.005	50.735	0.000	−29.640
	L	14.950	1.359	12.286	17.614					L	70.010	13.016	44.498	95.522		
	Overall	20.930	2.318	16.387	25.473					Overall	55.650	7.925	40.116	71.184		
*SPPL2A*	H	27.200	5.000	17.399	37.001	0.005	11.790		*TRPV1*	H	80.680	7.672	65.642	95.718	0.002	35.610
	L	15.410	2.331	10.842	19.978					L	45.070	6.030	33.250	56.890		
	Overall	20.930	2.318	16.387	25.473					Overall	55.650	7.925	40.116	71.184		
*TRPV1*	H	29.660	6.127	17.652	41.668	0.005	13.530									
	L	16.130	1.962	12.284	19.976											
	Overall	20.930	2.318	16.387	25.473											
*TUSC1*	H	15.740	2.003	11.814	19.666	0.001	−18.160									
	L	33.900	8.193	17.841	49.959											
	Overall	20.930	2.318	16.387	25.473											

#### Three-Gene-Combination of Prediction for OS in Liver Cancer

Similarly, three-gene-combinations of the 28 single genes significantly associated with OS confirmed by the single gene survival analyses and the multivariate analysis were formed, resulting in 3,276 three-gene-combinations. 930 of these 3,276 combinations were screened out on the conditions that the number of cases in both the co-high and co-low expression groups was > 25, and the *p*-values were < 0.01 according to the OS-related K-M analyses results.

Furthermore, 12 of the above 28 single genes that were noted to have an unknown association with liver cancer prognosis formed 220 three-gene-combinations. Out of the 220 combinations, there were 31 combinations in which the number of cases in both the co-high and co-low expression groups was > 25 and the OS-related survival analyses results showed *p* < 0.01.

**Figure 4:**
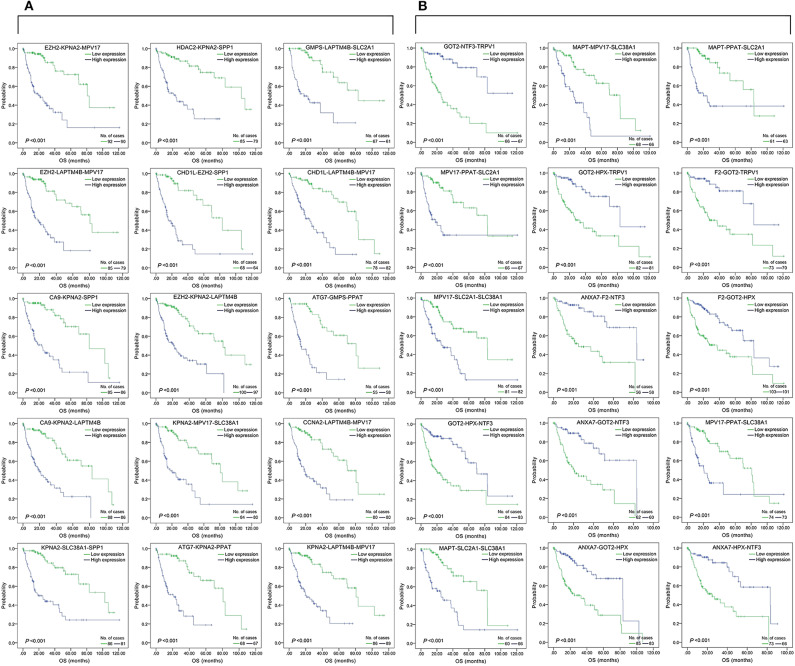
Association of the top 15 three-gene-combinations with smallest *p*-values with OS, using the data of HCC samples in a TCGA cohort and assessed by Kaplan-Meier analyses. The high expression group (blue line) of the combination consisted of samples with high expression of all three genes, and the low expression group (green line) of the combination consisted of samples with low expression of all three genes. The number of high and low expression groups in each combination was >25. **(A)** Association of OS and the top 15 combinations with the smallest *p*-values of the overall genes combinations. **(B)** Association of OS and the top 15 combinations with the smallest *p*-values of the unreported genes combinations.

**Figure 5:**
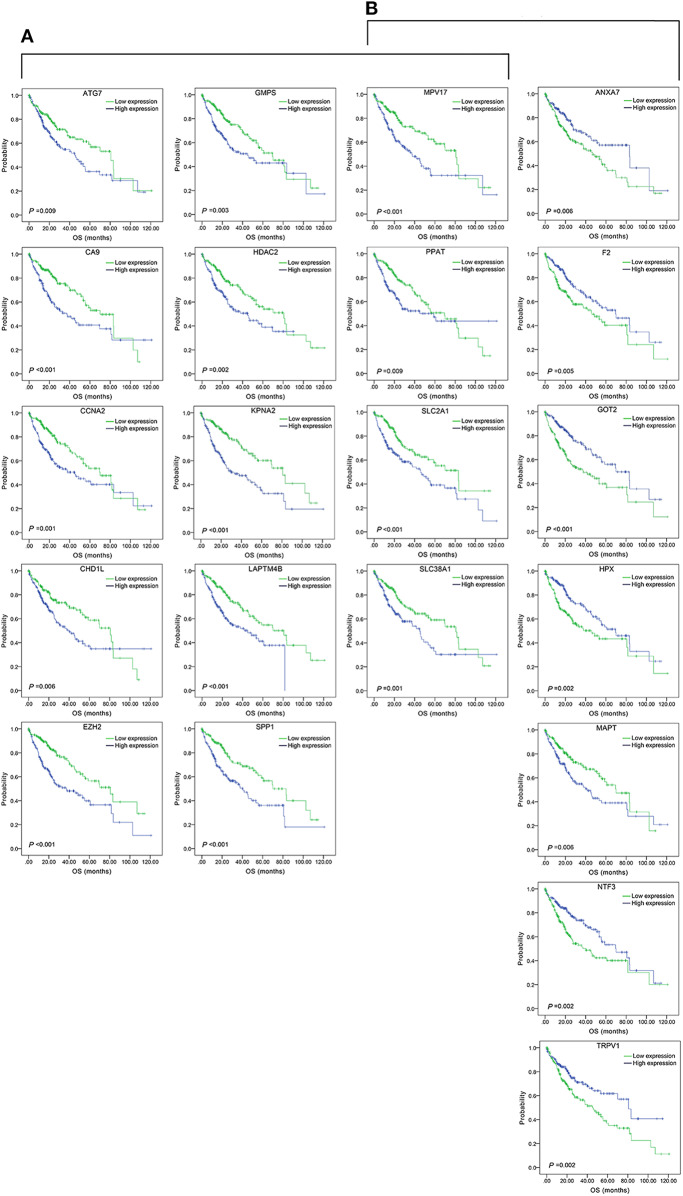
Association of OS and the individual genes contained in the top 15 combinations with the lowest *P*-values, using the data of HCC samples in a TCGA cohort and assessed by Kaplan-Meier analyses. **(A)** Association of OS and the 14 single genes contained in the first 15 total-gene combinations. **(B)** Association of OS and the 11 single genes contained in the first 15 unreported-gene combinations.

We found 930 of above 3,276 combinations and 31 of above 220 unreported-gene combinations were significant association with OS related survival of liver cancer patients. Among the 930 combinations and 31 combinations mentioned above, the diagrams and tables of the OS-related survival analyses of the top 15 combinations with the smallest *p*-values and the single genes they contain are as follows (Figures 4, 5; Tables 3, 4) Among the 12 genes that have an unknown association with HCC prognosis, *F2, GOT2, TRPV1*, and their combination *F2-GOT2-TRPV1* were all significantly associated with OS in 370 liver cancer samples from the TCGA data (*F2*: *p* = 0.005; *GOT2*: *p* < 0.001; *TRPV1*: *p* = 0.002; *F2-GOT2-TRPV1*: *p* < 0.001). The overall survival rate in HCC patients with low expression of *F2, GOT2, TRPV1*, and the three-gene-combination *F2*-*GOT2*-*TRPV1* were all significantly lower than that in liver cancer patients with high expression. In addition, the median survival time difference between the high expression group and the low expression group of *F2, GOT2, TRPV1*, and the three-gene combination *F2*-*GOT2*-*TRPV1* was 23.62, 32.26, 35.61, and 55.68 months, respectively. The median survival time difference of this combination was greater than that of a single gene, which was one of the main reasons why we selected these three genes for qRT-PCR and immunohistochemically validation.

**Table 4:** The associations of three-gene combinations with overall survival (OS) of HCC patients in a TCGA cohort, analyzed by Kaplan-Meier method.

**OS (Median) of combinations of 28 genes with HCC prognosis**									**OS (Median) of combinations of 12 genes have unknown association with HCC prognosis**							
		**Estimate**	**Std. Error**	**95% confidence interval**		***P***	**Median survival time difference (H-L)**				**Estimate**	**Std. Error**	**95% confidence interval**		***P***	**Median survival time difference (H-L)**
				**Lower boundary**	**Upper boundary**								**Lower boundary**	**Upper boundary**		
*EZH2-KPNA2-MPV17*	H	21.320	6.143	9.280	33.360	0.000	−59.360		*GOT2-NTF3-TRPV1*	H	–	–	–	–	0.000	–
	L	80.680	7.061	66.841	94.519					L	25.230	3.764	17.852	32.608		
	Overall	55.350	13.443	29.001	81.699					Overall	60.840	15.622	30.220	91.460		
*EZH2-LAPTM4B-MPV17*	H	18.230	5.735	6.988	29.472	0.000	−62.450		*MPV17-PPAT-SLC2A1*	H	18.330	4.916	8.695	27.965	0.000	−64.850
	L	80.680	7.990	65.020	96.340					L	83.180	15.794	52.224	114.136		
	Overall	48.950	10.014	29.323	68.577					Overall	53.350	15.422	23.123	83.577		
*CA9-KPNA2-SPP1*	H	23.780	5.368	13.259	34.301	0.000	−59.400		*MPV17-SLC2A1-SLC38A1*	H	25.130	9.272	6.957	43.303	0.000	−58.050
	L	83.180	16.292	51.248	115.112					L	83.180	7.322	68.829	97.531		
	Overall	51.250	11.668	28.381	74.119					Overall	46.750	6.571	33.870	59.630		
*CA9-KPNA2-LAPTM4B*	H	19.740	3.699	12.490	26.990	0.000	−63.440		*GOT2-HPX-NTF3*	H	70.010	10.631	49.174	90.846	0.000	50.430
	L	83.180	20.669	42.669	123.691					L	19.580	6.243	7.343	31.817		
	Overall	46.750	6.141	34.715	58.785					Overall	55.350	6.783	42.055	68.645		
*KPNA2-SLC38A1-SPP1*	H	19.090	6.876	5.614	32.566	0.000	−83.570		*MAPT-SLC2A1-SLC38A1*	H	25.130	9.134	7.227	43.033	0.000	−58.050
	L	102.660	21.958	59.622	145.698					L	83.180	12.085	59.493	106.867		
	Overall	69.510	10.951	48.047	90.973					Overall	45.890	7.002	32.167	59.613		
*HDAC2-KPNA2-SPP1*	H	23.780	5.613	12.778	34.782	0.000	−78.880		*MAPT-MPV17-SLC38A1*	H	25.130	4.047	17.197	33.063	0.000	−58.050
	L	102.660	14.189	74.850	130.470					L	83.180	9.720	64.128	102.232		
	Overall	83.180	21.672	40.704	125.656					Overall	45.070	6.284	32.754	57.386		
*CHD1L-EZH2-SPP1*	H	15.410	4.012	7.547	23.273	0.000	−67.770		*GOT2-HPX-TRPV1*	H	83.180	11.770	60.111	106.249	0.000	50.160
	L	83.180	9.866	63.842	102.518					L	33.020	6.971	19.356	46.684		
	Overall	46.750	12.305	22.633	70.867					Overall	70.010	14.673	41.251	98.769		
*EZH2-KPNA2-LAPTM4B*	H	21.680	5.445	11.008	32.352	0.000	−59.000		*ANXA7-F2-NTF3*	H	83.510	15.702	52.734	114.286	0.000	58.640
	L	80.680	7.011	66.939	94.421					L	24.870	10.561	4.170	45.570		
	Overall	46.750	10.389	26.388	67.112					Overall	53.350	14.230	25.459	81.241		
*KPNA2-MPV17-SLC38A1*	H	17.580	5.820	6.172	28.988	0.000	−63.100		*ANXA7-GOT2-NTF3*	H	83.180	23.271	37.569	128.791	0.000	62.580
	L	80.680	7.992	65.015	96.345					L	20.600	5.417	9.983	31.217		
	Overall	53.350	11.888	30.049	76.651					Overall	48.950	7.670	33.916	63.984		
*ATG7-KPNA2-PPAT*	H	21.120	6.087	9.190	33.050	0.000	−59.560		*ANXA7-GOT2-HPX*	H	83.180	13.677	56.373	109.987	0.000	58.310
	L	80.680	10.953	59.212	102.148					L	24.870	8.505	8.200	41.540		
	Overall	45.530	11.839	22.325	68.735					Overall	53.290	13.890	26.066	80.514		
*GMPS-LAPTM4B-SLC2A1*	H	17.970	6.680	4.876	31.064	0.000	−65.210		*MAPT-PPAT-SLC2A1*	H	20.110	6.433	7.501	32.719	0.000	−63.070
	L	83.180	16.018	51.784	114.576					L	83.180	14.580	54.602	111.758		
	Overall	53.350	13.670	26.557	80.143					Overall	70.010	18.751	33.258	106.762		
*CHD1L-LAPTM4B-MPV17*	H	24.870	4.882	15.302	34.438	0.000	−55.810		*F2-GOT2-TRPV1*	H	83.180	11.976	59.707	106.653	0.000	55.680
	L	80.680	7.912	65.172	96.188					L	27.500	6.805	14.162	40.838		
	Overall	55.650	10.709	34.660	76.640					Overall	81.670	20.419	41.649	121.691		
*ATG7-GMPS-PPAT*	H	13.960	4.451	5.236	22.684	0.000	−66.720		*F2-GOT2-HPX*	H	83.180	6.650	70.146	96.214	0.000	45.890
	L	80.680	17.665	46.057	115.303					L	37.290	7.225	23.129	51.451		
	Overall	37.680	8.510	21.001	54.359					Overall	69.510	12.170	45.657	93.363		
*CCNA2-LAPTM4B-MPV17*	H	18.330	3.559	11.354	25.306	0.000	−51.680		*MPV17-PPAT-SLC38A1*	H	20.600	5.930	8.977	32.223	0.000	−60.080
	L	70.010	6.190	57.878	82.142					L	80.680	9.365	62.324	99.036		
	Overall	48.950	7.272	34.697	63.203					Overall	51.250	13.888	24.030	78.470		
*KPNA2-LAPTM4B-MPV17*	H	21.320	5.082	11.359	31.281	0.000	−59.360		*ANXA7-HPX-NTF3*	H	83.180	26.573	31.096	135.264	0.000	58.310
	L	80.680	7.900	65.196	96.164					L	24.870	7.244	10.672	39.068		
	Overall	51.250	14.898	22.050	80.450					Overall	48.950	5.919	37.350	60.550		

### Low Expression of *F2, GOT2*, and *TRPV1* Predicts Poor Prognosis

Based on the above results of the OS-related survival analyses and multivariate analyses on 28 genes, as well as the results of survival analyses on their three-gene-combinations, we selected three genes *F2, GOT2*, and *TRPV1* with strong liver cancer prognostic potential for subsequent validation.

#### *F2, GOT2*, and *TRPV1* Were Downregulated in HCC Tissues

**Figure 6:**
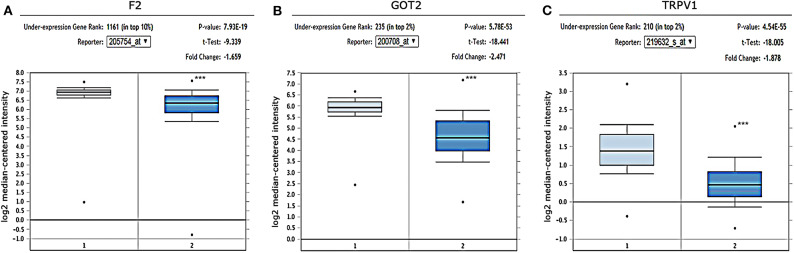
Expression of *F2, GOT2*, and *TRPV1* in HCC and adjacent normal liver tissues confirmed by independent microarrays from the Oncomine database. The expression of **(A)** *F2*, **(B)** *GOT2*, and **(C)** *TRPV1* were all significantly reduced in HCC tissues by the Roessler Liver 2 Statistics [225 HCC tissues (dark blue) vs. 220 normal liver tissues (light blue)]. ***p < 0.001.

The gene expression in HCC was determined based on three independent microarrays which are all collected in Oncomine database (https://www.oncomine.org/resource/login.html). As shown in Roessler Liver 2 Statistics (225 HCC tissues vs. 220 liver tissues), the expression of *F2, GOT2*, and *TRPV1* in HCC tissues were all significantly down-regulated compared with that in normal liver tissues. (*p* < 0.001; Figure 6) In addition, based on the Mas Liver Statistics (38 HCC tissue vs. 19 liver tissue), both *F2* and *TRPV1* were significantly down-regulated in HCC tissues. Based on the Chen Liver Statistics (104 HCC tissues vs. 76 liver tissues), both *F2* and *GOT2* were significantly down-regulated in HCC tissues.

**Figure 7:**
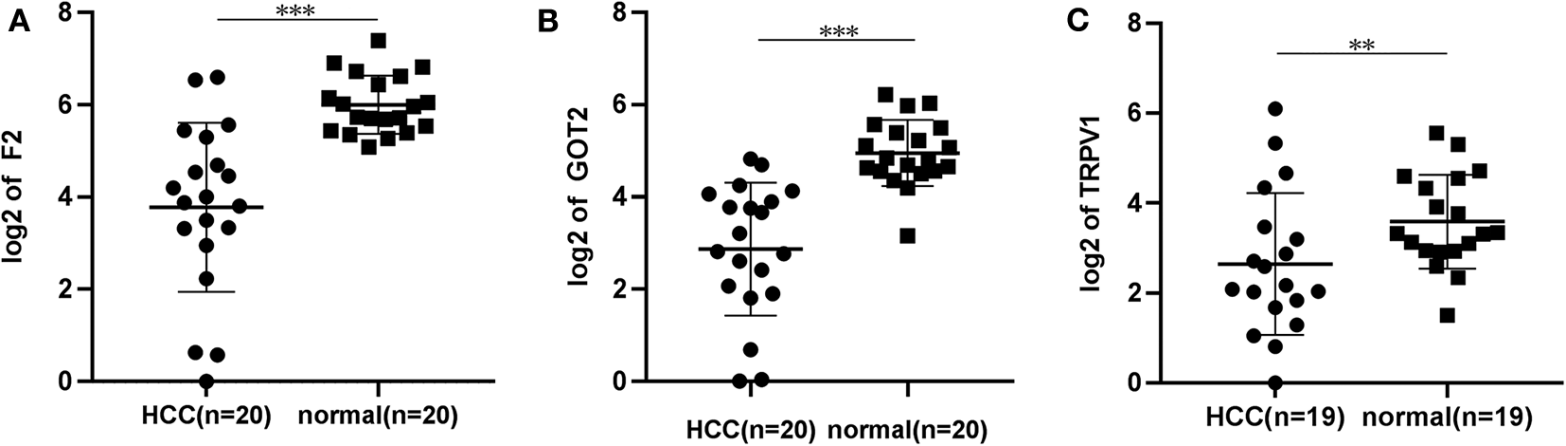
The relative expression levels of *F2, GOT2*, and *TRPV1* were assessed by qRT-PCR in HCC tissues and adjacent liver tissues. **(A)** The expression levels of *F2* were assessed by qRT-PCR in 20 pairs of HCC tissues and peritumoral tissues. Paired *t*-test, ***p < 0.001. **(B)** The relative expression levels of *GOT2* were assessed by qRT-PCR in 19 pairs of HCC tissues and peritumoral tissues. Paired *t*-test, ***p < 0.001. **(C)** The relative expression levels of *TRPV1* were assessed by qRT-PCR in 19 pairs of HCC tissues and peritumoral tissues. Paired *t*-test, log, **p < 0.01.

The qRT-PCR results of *F2, GOT2* and *TRPV1* showed that 20/20, 19/20, and 16/19 of the HCC tissues exhibited significantly lower expression of *F2* (*p* < 0.001; Figure 7A)*, GOT2* (*p* < 0.001; Figure 7B), and *TRPV1* (*p* = 0.006; Figure 7C), respectively, when compared with their corresponding non-tumorous tissues.

**Figure 8:**
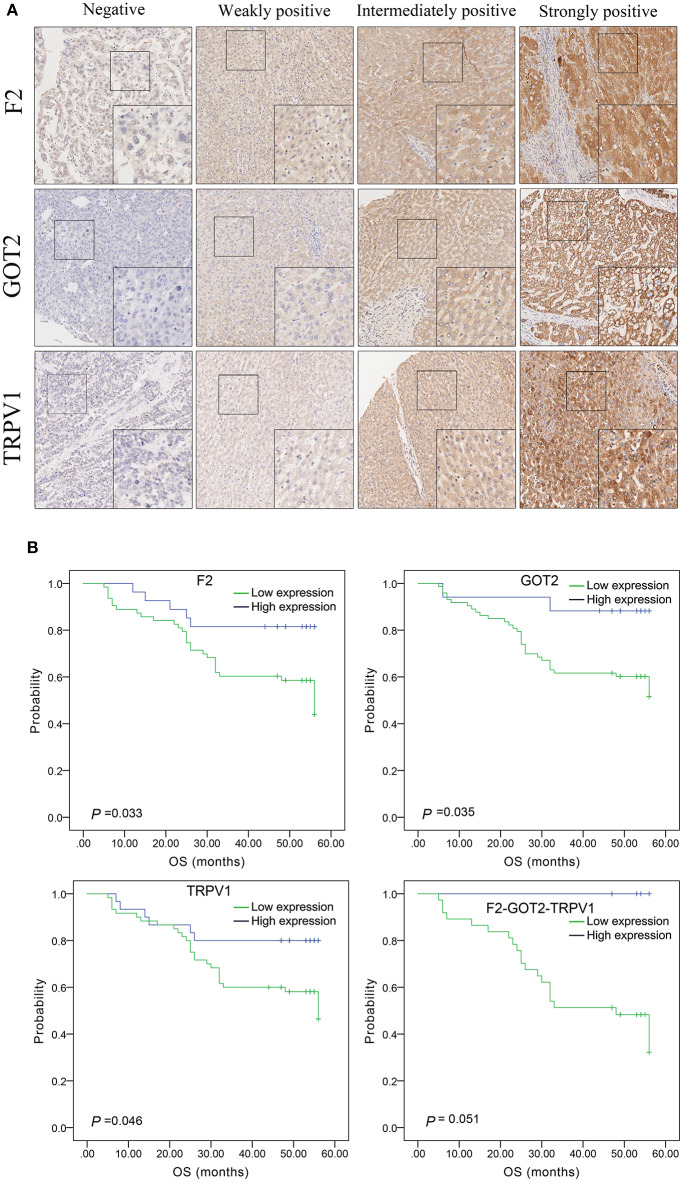
The expression of *F2, GOT2*, and *TRPV1* in 90 pairs of HCC and adjacent normal liver tissues of biological tissue microarray by IHC, and the association with HCC patients prognosis. **(A)** Negative, weakly positive, intermediately positive, and strongly positive IHC staining of *F2, GOT2*, and *TRPV1*. *F2, GOT2*, and *TRPV1* were all low expressed in liver cancer. **(B)** The lower protein expression levels of *F2, GOT2*, and *TRPV1* were all associated with 5-year OS of 90 HCC patients, examing by Kaplan-Meier analyses and log-rank test. However, there was marginally significant association between the *F2*-*GOT2*-*TRPV1* combination protein expression levels with the OS of HCC patients. (*F2*: *p* = 0.033, *GOT2*: *p* = 0.035, *TRPV1*: *p* = 0.046, *F2*-*GOT2*-*TRPV1: p* = 0.051).

The protein expression of *F2, GOT2*, and *TRPV1* in HCC tissues was evaluated using IHC. Positive staining of *F2, GOT2*, and *TRPV1* was mainly localized in the cytoplasm of HCC cells. The representative staining of *F2, GOT2*, and *TRPV1* negative and positive protein expression in HCC are shown in Figure 8A.

Among 90 HCC tissues and adjacent non-malignant liver tissues, IHC was employed to measure the protein expression of *F2, GOT2*, and *TRPV1*, respectively. Low *F2* expression was observed in 62/89 (69.66%) of the HCC tissues, compared to 33/89 (37.08%) in adjacent normal liver tissues (*p* < 0.001); low *GOT2* expression was noted in 72/89 (80.90%) of the HCC tissues, compared to 32/89 (35.96%) in adjacent normal liver tissues (*p* < 0.001); low *TRPV1* expression was also observed in 59/89 (66.29%) of the HCC tissues, compared to 38/89 (42.70%) in adjacent normal liver tissues (*p* = 0.002).

#### Expression of *F2, GOT2*, and *TRPV1* and Their Combination *F2-GOT2-TRPV1* With OS

Based on the above results of single-genes and three-gene combinations survival analyses of TCGA HCC samples, the low expression of *F2, GOT2, TRPV1* and their combination *F2-GOT2-TRPV1* was significantly associated with poor OS in HCC. (*F2*: *p* = 0.005; *GOT2*: *p* < 0.001; *TRPV1*: *p* = 0.002; *F2-GOT2-TRPV1*: *p* < 0.001). In addition, the median survival time difference between the high expression group and the low expression group of *F2*-*GOT2*-*TRPV1* was greater than that of any of the three single genes.

The results of IHC for 90 liver cancer cases showed that the low protein expression of *F2, GOT2*, and *TRPV1* was significantly associated with lower 5-year survival in HCC patients (*F2*: *p* = 0.033, *GOT2*: *p* = 0.035, *TRPV1*: *p* = 0.046; K-M survival analyses). However, due to the insufficient number of events in the co-high expression group of the combination *F2-GOT2-TRPV1*, there was marginally significant difference found in the overall survival rate of HCC patients between the co-high expression group and the co-low expression group of the protein combination *F2-GOT2-TRPV1* (*p* = 0.051) (Figure 8B).

## Discussion

Liver cancer is characterized by inconspicuous early symptoms, a high degree of malignancy, recurrence and spread, and unsatisfactory prognosis. With limited treatment options, it is one of the common malignancies that plague the world. Therefore, identification of effective prognostic biomarkers for liver cancer is the key to improving the efficacy of targeted therapy for HCC and reducing the adverse prognostic effects of liver cancer.

In our study, by combining and searching 15 corresponding concepts of the key words “liver cancer,” “prognosis,” and “outcome,” and according to *p*-values < 0.05, 1,173 genes that may be related to the prognosis of liver cancer were mined from the Coremine platform after merging and removing duplicates. However, due to the insufficient sample size and data related to the prognosis of liver cancer in the Coremine platform as well as the large heterogeneity among the samples, we also selected gene expression data and prognosis data of 319 samples for DFS and 370 samples for OS from the TCGA platform. We then separately conducted DFS-related and OS-related K-M survival analysis for each gene, followed by multivariate analyses, respectively. The large-scale genes mining and a large number of homogenous samples gave us a reliable analytical foundation. By far, this is the first large-scale survival analyses for hundreds of genes for subsequent screening.

In addition, the genes selected by K-M survival analyses with a low *p*-value (*p* < 0.01) were further screened by multivariate analyses using the Cox proportional hazards regression model. We found that 39 genes and 28 genes were reliably and significantly associated with DFS and OS, respectively, in liver cancer. Many of the above genes have been confirmed to be associated with the prognosis of HCC by previous reports. For example, of the 39 DFS-related genes, *ALDOB* inhibits metastasis in HCC and can be a valuable novel prognosis predicting marker (30); *APOB* was found to be a prognostic biomarker for patients with radical resection of HCC (31, 32); *CCNF* is downregulated in HCC and is a promising prognostic marker (33). In addition, *CPT2* (34), *G6PD* (35), *GNMT* (36), *NEK2* (37), etc. have also been reported to be prognostic markers of HCC by affecting the occurrence or invasion of HCC. The above findings are consistent with what we identified. Other genes, such as *C5, CD4, CETP, COL18A1, DAND5, DNASE1, EBPL, F7, FLT3, ITGB2, KNG1, LMOD1, PPAT, PPIA, PRF1, SELP, SPPL2A*, and *TRPV1* that have not been systematically reported in relation to the prognosis of liver cancer, are our newly discovered prognostic markers for DFS in liver cancer. Similarly, of the 28 OS-related genes, *CA9* regulates the epithelial-mesenchymal transition and is a novel prognostic marker in HCC (38), *E2F1* expression has an impact on tumor aggressiveness and affects the prognosis of HCC (14, 15), *CYP3A4* (39), *HDAC2* (40), and *KPNA2* (41) have also been identified as prognostic markers of HCC and are reflected in our findings. The other genes, such as *ANXA7, F2, GOT2, HPX, MAGEB3, MAPT, MPV17, NTF3, PPAT, SLC2A1, SLC38A1*, and *TRPV1* are all novel prognostic markers associated with liver cancer OS found by our reliable and large-scale screening studies. Three genes (*APOB, PPAT*, and *TRPV1*) were associated with both DFS and OS of HCC, suggesting that *APOB, PPAT*, and *TRPV1* may be significant and effective in predicting both the progress and the adverse outcomes of HCC.

Moreover, there may be connections among the above selected genes and they can work together to influence the development and prognosis of liver cancer to some extent. Although there are some genes that had been reported as prognostic molecular markers of liver cancer, most reports focused on the impact of a single gene on the prognosis of liver cancer, few studies performed such a large-scale survival analysis. Studies of multiple gene combinations are more effective than the analysis of single genes in predicting the prognosis of liver cancer.

In our study, we performed three-gene combinations of the 39 DFS-related genes and 28 OS-related genes screened from the above survival analyses. In order to further study the predictive effect of the combinations constituted by the selected genes on the prognosis of liver cancer, and to compare the predictive power of single genes and corresponding gene combinations, we carried out thousands of K-M survival analyses on these combinations. To ensure the comparability and credibility, we removed the combinations of which the co-high or co-low expression group cases were fewer than 26, and screened 2,758 DFS-related combinations and 930 OS-related combinations with *p*-values < 0.01. Moreover, we also performed three-gene-combination models and K-M survival analyses on the 18 DFS-related genes and 12 OS-related genes we found but have not been systematically reported to be related to the prognosis of HCC. 317 unreported-gene combinations and 31 unreported-gene combinations significantly associated with DFS and OS, respectively, were screened out.

For the above four types of three-gene-combinations (the overall genes combinations associated with DFS, the unreported genes combinations associated with DFS, the overall genes combinations associated with OS, and the unreported genes combinations associated with OS), the top 15 combinations with the lowest *p*-values of the survival analyses and the genes they contained were, respectively, selected for comparison (Tables 2, 3, 4).

For example, for the overall gene combinations associated with OS, *KPNA2-SLC38A1-SPP1*, the median survival time difference between the co-high and the co-low expression group was 83.57 months. In contrast, that of the single genes *KPNA2, SLC38A1*, and *SPP1*, was 47.66, 35.61, and 29.64 months, respectively. After combining *KPNA2, SLC38A1*, and *SPP1*, the median survival time difference between the high and low expression groups was larger than that of any of the three single genes by at least 36 months. This shows that these three genes *KPNA2, SLC38A1*, and *SPP1*, after combination, may be better predictive values for liver cancer prognosis and may be more clinically useful for future treatment target selection.

We also selected genes that have not been previously reported for liver cancer prognosis and compared their prognostic efficacy with the corresponding three-gene combinations (the chart only shows the top 15 groups with the lowest *p*-values of the three-gene combinations prognostic models). The expression of one of the combinations *F2-GOT2-TRPV1* had a greater effect on the median survival time of OS than any of the three individual genes (The median survival time difference: *F2-GOT2-TRPV1*: 55.68 months; *F2*: 23.62 months; *GOT2*: 32.26 months; *TRPV1*: 35.61 months).

Coagulation factor II (*F2*) plays a major role in proteolysis to form thrombin in the first step of the coagulation cascade and eventually generates hemostasis. An enrichment analysis of genetic changes during the development of HCC identified several hub genes, including *F2*, which interacts in several groups of conditional specific PPI networks (42). Additionally, it was reported that *F2* is associated with invasion in neuroendocrine prostate cancer (43). Glutamic-oxaloacetic transaminase 2 (*GOT2*) plays an important role in amino acid metabolism and the tricarboxylic acid cycle, and it affects the malate-aspartic acid shuttle activity and glycolysis in the liver under the stimulation of liver inflammation. (44, 45) *TRPV1* is a regulator of cell homeostasis, previous studies have revealed that the expression of *TRPV1* is significantly decreased in renal cell carcinoma, colorectal cancer, and melanoma. In addition, *TRPV1* can affect P53 and *TRPV1*-dependent pathways to inhibit the growth of colorectal cancer and melanoma (46–48), and can cause apoptosis in human osteosarcoma MG63 cells (49).

At present, there are few studies on the above three genes *F2, GOT2, TRPV1* and particular their combinations in the prognosis of HCC. In our study, the results of the 20 pairs of HCC and paracancerous tissues for qRT-PCR, as well as 90 pairs HCC biochips for IHC confirmed that all of the *F2, GOT2*, and *TRPV1* genes are significantly and consistently down-expressed in HCC tissues, and this is reconfirmed by three independent microarrays. Moreover, the low expression of *F2, GOT2*, and *TRPV1* were all significantly associated with poor prognosis of HCC. However, due to the number of death events in the *F2-GOT2-TRPV1* high expression group of in the HCC biochips being 0, the survival analysis of the *F2-GOT2-TRPV1* high and the expression group was marginally significant (*p* = 0.051), but this is still consistent with our above-mentioned big data-based multi-gene combination survival analysis results.

As there may be certain relationships between the genes we screened that are significantly associated with the prognosis of liver cancer, they can work together in the form of multi-gene combinations in the development of liver cancer. However, the predictive potency of different gene combinations varies. Some combinations are better predictors than individual genes, and therefore these combinations may be more valuable than individual genes in determining the target site for liver cancer prognosis. Due to limitations in human and material resources, it still remains unclear how these genes and gene combinations specifically affect the HCC survival. Further investigation and experimentations are needed to elucidate the biological mechanisms of the selected genes, particularly for the significant multi-gene combinations, in the development and progression of HCC.

Our findings cover a large gene level, and we have also explored the predictive efficacy of a number of gene combinations for the prognosis of liver cancer. We believe that these highly significant prognostic-related genes and gene combinations derived from the above multiple screenings are promising, reliable molecular markers for the prognosis of liver cancer, and our screening methods can be extended to other tumor types.

In conclusion, based on a large sample size of public data platform, novel and effective data mining and multiple screening methods, large-scale survival analyses, as well as supplemental reliable experimental verification, we identified a series of novel genes and multi-gene combinations that are significantly associated with DFS or OS in liver cancer. Moreover, a huge difference between high and low expression group of these three-gene combination was detected. Some of the three-gene combinations can predict much longer or shorter survival time for liver cancer patients than the single genes. QRT-PCR, immunohistochemistry, and three independent microarray results confirmed our findings that three of the selected novel genes *F2, GOT2*, and *TRPV1*, as well as the corresponding combination *F2-GOT2-TRPV1*, showed significantly lower expression in HCC and are associated with OS in HCC. Some gene combinations may be more predictors of prognosis than single genes and can be used as potential effective therapeutic targets for liver cancer.

## Data Availability Statement

The datasets generated for this study are available on request to the corresponding author.

## Ethics Statement

The studies involving human participants were reviewed and approved by The Ethics Committee of Guangxi Medical University. The patients/participants provided their written informed consent to participate in this study.

## Author Contributions

ML and XLi performed most analysis. ML led the writing of the manuscript. SL provided the clinical samples and participated in revising the manuscript. FX and JT participated in drafting and reviewing the manuscript. EG conducted a search for genes and preliminary screening work by keyword. XQ obtained and matched the TCGA samples data. ML, LW, and QL performed the single-gene and multi-gene-combination survival analyses. ZL and LL conducted an inquiry about the relevant information of the selected genes. XLu performed validation of the selected genes in three microarrays. KL and DZ performed the mRNA isolation and qRT-qPCR, and collected and analyzed experimental data. YY and XLi were subjected to immunohistochemistry and experimental data processing. FY and XZ participated in designing and reviewing the study. All the authors reviewed the manuscript and all the authors read and approved the final manuscript.

## Conflict of Interest

The authors declare that the research was conducted in the absence of any commercial or financial relationships that could be construed as a potential conflict of interest.
