# Supplementary material for: Big Data-Based Identification of Multi-Gene Prognostic Signatures in Liver Cancer
Source: Front Oncol. 2020 May 28;10:847. doi: 10.3389/fonc.2020.00847 (PMC7270198; doi:10.3389/fonc.2020.00847)
Supplement: Supplementary file 1 [file Data_Sheet_1.docx]

Supplementary Material

| **Supplementary Table S1.** Keywords and their corresponding concepts for searching for their corresponding genes in the Coremine database | |
| --- | --- |
| Keywords | Corresponding concepts |
| "Liver cancer" | Liver neoplasms (alias Liver Cancer) (disease) (60666 connections) |
|  | Liver carcinoma (alias liver cell cancer) (disease) (55739 connections) |
|  | Carcinoma, Hepatocellular (alias Adult Liver Cancer) (mesh) (57034 connections) |
| "Prognosis" | Prognosis (mesh) (77312 connections) |
|  | Prognostic Marker (alias Prognosis Marker) (chemical) (22056 connections) |
| "Outcome" | Fatal Outcome (mesh) (34016 connections) |
|  | Outcome Assessment (Health Care) (alias Outcome Study) (mesh) (48296 connections) |
|  | Outcome studies (procedure) (9545 connections) |
|  | Treatment Outcome (mesh) (77246 connections) |
|  | Outcomes research (procedure) (5540 connections) |
|  | Outcome monitoring (procedure) (2030 connections) |
|  | Patient-focused outcomes (procedure) (3830 connections) |
|  | Treatment outcome in HSR (procedure) (998 connections) |
|  | Patient Reported Outcome Measures (alias Patient Reported Outcome) (mesh) (2301 connections) |
|  | Patient Outcome Assessment (mesh) (9066 connections) |

| **Supplementary Table S2.** 1173 candidate genes that may be related to the prognosis of HCC retrieved in Coremine with P < 0.05 as the bound | | | | | | | | | | | | | | |
| --- | --- | --- | --- | --- | --- | --- | --- | --- | --- | --- | --- | --- | --- | --- |
| 1173 candidate genes | | | | | | | | | | | | | | |
| *A2M* | *ASCC1* | *CCL20* | *CLU* | *DLK1* | *FLT1* | *GSTM2* | *IL1A* | *LIFR* | *MTA1* | *PAK1* | *PRKAA1* | *SATB1* | *SST* | *TRADD* |
| *ABCA1* | *ASGR1* | *CCL4* | *CNBP* | *DNAH8* | *FLT3* | *GSTP1* | *IL1B* | *LILRB1* | *MTDH* | *PAK3* | *PRKAA2* | *SCARB1* | *SSTR2* | *TRAF2* |
| *ABCA3* | *ASS1* | *CCL4L1* | *CNR1* | *DNAJB1* | *FLT4* | *GTF3A* | *IL2* | *LIN28A* | *MTOR* | *PARP1* | *PRKAB1* | *SCARB2* | *STARD13* | *TRH* |
| *ABCB1* | *ATAD2* | *CCNA2* | *COL11A2* | *DNASE1* | *FN1* | *GUSB* | *IL22* | *LIN28B* | *MTSS1* | *PAX5* | *PRKCA* | *SCD* | *STAT1* | *TRIB3* |
| *ABCB11* | *ATAT1* | *CCNB1* | *COL18A1* | *DNMT1* | *FOS* | *GZMB* | *IL24* | *LIPC* | *MTTP* | *PCK1* | *PRKCSH* | *SCO2* | *STAT2* | *TRIM26* |
| *ABCB4* | *ATF2* | *CCND2* | *COMMD7* | *DNMT3A* | *FOSB* | *H2AFX* | *IL27* | *LMLN* | *MUC1* | *PCK2* | *PRKDC* | *SCT* | *STAT3* | *TRPV1* |
| *ABCC1* | *ATF3* | *CCNE1* | *COPS5* | *DNMT3B* | *FOXA1* | *HAMP* | *IL2RA* | *LMNA* | *MUC16* | *PCLAF* | *PRKN* | *SDC1* | *STAT4* | *TSC1* |
| *ABCC2* | *ATF4* | *CCNF* | *CORO1A* | *DPP4* | *FOXA2* | *HDAC1* | *IL2RG* | *LMNB1* | *MUC2* | *PCNA* | *PRL* | *SDC2* | *STAT5A* | *TSPAN1* |
| *ABCC3* | *ATF5* | *CCNG1* | *CORO7* | *DPYD* | *FOXF1* | *HDAC2* | *IL3* | *LMOD1* | *MUL1* | *PCSK9* | *PRNP* | *SDHC* | *STAT5B* | *TSPO* |
| *ABCG2* | *ATF6* | *CCR6* | *CP* | *DR1* | *FOXM1* | *HDAC3* | *IL4* | *LPAR1* | *MVP* | *PDCD1* | *PROC* | *SELENOP* | *STK11* | *TTC23* |
| *ACAN* | *ATF6B* | *CD151* | *CPQ* | *DUSP1* | *FOXO1* | *HDAC9* | *IL6* | *LPAR2* | *MX1* | *PDCD4* | *PROM1* | *SELL* | *STK4* | *TTF1* |
| *ACAT1* | *ATG5* | *CD177* | *CPT2* | *DUSP21* | *FOXO3* | *HDGF* | *IL6R* | *LPL* | *MXI1* | *PDE5A* | *PROX1* | *SELP* | *SUB1* | *TTR* |
| *ACE* | *ATG7* | *CD1A* | *CR1* | *DYNLL1* | *FOXP3* | *HES1* | *IL6ST* | *LRPPRC* | *MYCN* | *PDGFC* | *PRTN3* | *SERPINA1* | *SUGP1* | *TUSC1* |
| *ACHE* | *ATIC* | *CD274* | *CRAT* | *E2F1* | *FPR1* | *HFE* | *IMP3* | *MACC1* | *MYD88* | *PDGFRA* | *PSG1* | *SERPINA3* | *SULF1* | *TWIST1* |
| *ACKR3* | *ATM* | *CD276* | *CREB1* | *E2F2* | *FRZB* | *HFM1* | *INHBE* | *MAD2L1* | *MYOG* | *PDGFRB* | *PSMC4* | *SERPINA7* | *SULF2* | *TXK* |
| *ACO1* | *ATOH7* | *CD33* | *CREBBP* | *E2F3* | *FSHR* | *HGF* | *INS* | *MAGEA1* | *NAA50* | *PDIA3* | *PTBP1* | *SERPINB2* | *SULT1A1* | *TXN* |
| *ACOX1* | *ATP12A* | *CD34* | *CREM* | *EBAG9* | *FST* | *HIF1A* | *INSM1* | *MAGEA3* | *NANOG* | *PDIK1L* | *PTDSS2* | *SERPINB3* | *SULT1C4* | *TXNRD1* |
| *ACP1* | *ATP4A* | *CD36* | *CRH* | *EBPL* | *FTH1* | *HIST2H2AA3* | *INSR* | *MAGEA4* | *NAT2* | *PDK1* | *PTEN* | *SERPINB5* | *SULT1E1* | *TYK2* |
| *ACPP* | *ATP6V0D1* | *CD38* | *CRP* | *ECHS1* | *FUNDC2* | *HIST2H2AC* | *IQGAP1* | *MAGEB3* | *NBR1* | *PDK4* | *PTGS2* | *SERPINC1* | *SULT2A1* | *TYMP* |
| *ACTA2* | *ATP7B* | *CD4* | *CRYZ* | *EDN1* | *FURIN* | *HK1* | *IQGAP2* | *MAGI1* | *NCAM1* | *PDLIM5* | *PTH* | *SERPINE1* | *SUMF2* | *TYMS* |
| *ACTB* | *AURKAIP1* | *CD40* | *CSF3* | *EEF1A2* | *FUT4* | *HK2* | *IRF1* | *MALAT1* | *NCL* | *PDPK1* | *PTHLH* | *SERPINF1* | *SYCE1L* | *TYR* |
| *ADAM10* | *AURKB* | *CD44* | *CSNK1A1L* | *EFNA1* | *FUT8* | *HMGA1* | *IRF3* | *MAP1LC3A* | *NCOA1* | *PDYN* | *PTK2* | *SERPING1* | *SYP* | *UBASH3B* |
| *ADAM17* | *AVP* | *CD53* | *CSRP1* | *EGF* | *FZD1* | *HMGA2* | *IRF9* | *MAP1LC3B* | *NDRG1* | *PDZD2* | *PTP4A3* | *SETD1A* | *SYT1* | *UBD* |
| *ADAM9* | *AXIN1* | *CD68* | *CTAG1B* | *EGFL7* | *FZD7* | *HMGB1* | *IRS1* | *MAP2K1* | *NDUFAF1* | *PEG10* | *PTPA* | *SETD2* | *TAC1* | *UBE2B* |
| *ADH1B* | *AXIN2* | *CD7* | *CTGF* | *EGFR* | *FZR1* | *HMGCR* | *IRS2* | *MAP2K4* | *NDUFAF2* | *PEMT* | *PTPN1* | *SFRP1* | *TANK* | *UBQLN3* |
| *ADH1C* | *BACE1* | *CD79A* | *CTLA4* | *EGR1* | *G6PC* | *HMOX1* | *ISG20* | *MAP2K7* | *NEDD9* | *PENK* | *PTPN11* | *SHBG* | *TAP1* | *UCA1* |
| *ADIPOQ* | *BAD* | *CD80* | *CTNNA1* | *EHHADH* | *G6PD* | *HNF1A* | *ITGA5* | *MAP3K1* | *NEK2* | *PER1* | *PTPN6* | *SHH* | *TAS2R13* | *UCK2* |
| *ADK* | *BAK1* | *CD81* | *CTNNB1* | *EIF2AK2* | *GAA* | *HNF1B* | *ITGA6* | *MAP3K5* | *NELFCD* | *PFDN5* | *PTPRC* | *SIRT1* | *TAT* | *UGT1A1* |
| *AFP* | *BAX* | *CD83* | *CTSB* | *EIF2S1* | *GABPA* | *HNF4A* | *ITGAL* | *MAP3K7* | *NELFE* | *PGAP3* | *PXN* | *SIRT3* | *TBC1D9* | *UGT1A6* |
| *AGO2* | *BBC3* | *CD86* | *CTSD* | *EIF4E* | *GABRP* | *HNRNPC* | *ITGAM* | *MAPK1* | *NET1* | *PGF* | *QRSL1* | *SKP2* | *TCEAL1* | *UOX* |
| *AGT* | *BBS9* | *CD99L2* | *CTTN* | *EIF4EBP1* | *GADD45A* | *HNRNPDL* | *ITGAV* | *MAPK14* | *NF1* | *PGK1* | *QSOX1* | *SLC10A1* | *TCF21* | *URGCP* |
| *AGTR1* | *BCL2* | *CDC123* | *CUL4A* | *EIF5A* | *GADD45B* | *HOTAIR* | *ITGB1* | *MAPK3* | *NFE2* | *PHB* | *RAB5A* | *SLC12A9* | *TCF3* | *USF1* |
| *AHR* | *BCL2L1* | *CDC20* | *CUX1* | *EIF5A2* | *GADD45G* | *HP* | *ITGB2* | *MAPK8* | *NFE2L2* | *PIK3CA* | *RAC1* | *SLC1A5* | *TCF4* | *USP22* |
| *AICDA* | *BDNF* | *CDC25A* | *CXCL10* | *ELAVL1* | *GALNT14* | *HPDL* | *ITGB4* | *MAPK9* | *NFKB1* | *PIM2* | *RACK1* | *SLC22A1* | *TCF7L2* | *VEGFA* |
| *AIFM1* | *BECN1* | *CDC25B* | *CXCL12* | *ELK1* | *GALNT5* | *HPGDS* | *ITIH4* | *MAPT* | *NFKB2* | *PIN1* | *RAD51* | *SLC25A13* | *TCHP* | *VEGFC* |
| *AIP* | *BGLAP* | *CDC25C* | *CXCL6* | *ELN* | *GAPDH* | *HPRT1* | *ITPA* | *MARK2* | *NFKBIA* | *PIWIL2* | *RAET1E* | *SLC2A1* | *TERC* | *VIM* |
| *AKAP12* | *BHLHE40* | *CDC42* | *CXCL8* | *ENG* | *GAST* | *HPSE* | *JAG1* | *MARVELD1* | *NME1* | *PKLR* | *RAF1* | *SLC2A2* | *TERT* | *VIP* |
| *AKR1A1* | *BHMT* | *CDC6* | *CXCR1* | *ENO1* | *GBE1* | *HPX* | *JAK1* | *MAT1A* | *NME2* | *PKM* | *RALBP1* | *SLC2A3* | *TF* | *VLDLR* |
| *AKR1B10* | *BID* | *CDH1* | *CXCR4* | *ENO2* | *GCG* | *HRAS* | *JAK2* | *MAT2A* | *NOP53* | *PLA2G1B* | *RARA* | *SLC33A1* | *TFAM* | *VPS37A* |
| *AKR1C1* | *BIRC2* | *CDH17* | *CXXC1* | *ENPP2* | *GCK* | *HS6ST1* | *JUN* | *MB* | *NOS1* | *PLA2G4A* | *RARB* | *SLC38A1* | *TFAP2A* | *VTN* |
| *AKR1C2* | *BIRC3* | *CDH2* | *CYBB* | *EP300* | *GCLC* | *HSD17B6* | *JUND* | *MBD2* | *NOS2* | *PLAT* | *RASA1* | *SLC38A2* | *TFE3* | *WNK1* |
| *AKT1* | *BIRC5* | *CDK1* | *CYCS* | *EPCAM* | *GCLM* | *HSF1* | *KDR* | *MBP* | *NOS3* | *PLAU* | *RASSF1* | *SLC3A2* | *TFR2* | *WNT1* |
| *AKT2* | *BIRC7* | *CDK2* | *CYLD* | *EPHA2* | *GCNT2* | *HSP90AA1* | *KEAP1* | *MBTPS1* | *NOTCH1* | *PLAUR* | *RB1* | *SLC43A2* | *TFRC* | *WNT3* |
| *AKT3* | *BLZF1* | *CDK4* | *CYP19A1* | *EPHB4* | *GEMIN4* | *HSP90B1* | *KHDRBS1* | *MCL1* | *NOTCH2* | *PLB1* | *RBL2* | *SLC45A2* | *TG* | *WNT3A* |
| *ALB* | *BMI1* | *CDK5R2* | *CYP1A1* | *EPHX1* | *GFAP* | *HSPA1A* | *KIF1B* | *MCM3* | *NOTCH3* | *PLEC* | *RBM39* | *SLC5A5* | *TGFA* | *WT1* |
| *ALDH1A1* | *BMP6* | *CDK6* | *CYP1A2* | *EPO* | *GGH* | *HSPA4* | *KIT* | *MCM7* | *NOTCH4* | *PLG* | *RBMS3* | *SLC6A4* | *TGFB1* | *WWOX* |
| *ALDH2* | *BNIP3* | *CDK7* | *CYP1B1* | *EPX* | *GGTLC1* | *HSPA5* | *KLF4* | *MDK* | *NOX4* | *PLIN2* | *RBP4* | *SLC6A8* | *TGFBR1* | *WWTR1* |
| *ALDH3A1* | *BRAF* | *CDKN1A* | *CYP27B1* | *ERBB2* | *GH1* | *HSPA8* | *KLF6* | *MDM2* | *NPC2* | *PLK1* | *RECK* | *SLC7A1* | *TGFBR2* | *XAF1* |
| *ALDOB* | *BRCA1* | *CDKN1B* | *CYP2A6* | *ERCC1* | *GHR* | *HSPA9* | *KLHL1* | *MDM4* | *NPM1* | *PLPP5* | *REG3A* | *SLC9A6* | *TH* | *XBP1* |
| *ANGPT2* | *BSG* | *CDKN1C* | *CYP2B6* | *ERCC2* | *GHRH* | *HSPB1* | *KLK15* | *MEF2D* | *NPY* | *PLTP* | *RELA* | *SLCO1B1* | *THBS1* | *XPO4* |
| *ANKRD46* | *C3* | *CDKN2A* | *CYP2B7P* | *ERN1* | *GJA1* | *HSPB2* | *KLK3* | *MEG3* | *NPY4R* | *PLXNB2* | *REN* | *SLCO1B3* | *THPO* | *XRCC1* |
| *ANPEP* | *C5* | *CDKN2B* | *CYP2C9* | *ESM1* | *GJA8* | *HSPB3* | *KLK4* | *MEN1* | *NQO1* | *PMAIP1* | *RET* | *SLU7* | *THY1* | *XRCC5* |
| *ANXA1* | *CA1* | *CDKN2D* | *CYP2D6* | *ESR1* | *GJB1* | *HSPD1* | *KLRK1* | *MET* | *NR0B1* | *PML* | *RGN* | *SMAD2* | *TIA1* | *XRCC6* |
| *ANXA2* | *CA3* | *CDKN3* | *CYP2E1* | *ESRRB* | *GLB1* | *HSPG2* | *KNG1* | *MFN2* | *NR0B2* | *PNLIPRP3* | *RHAG* | *SMAD3* | *TICAM1* | *YAP1* |
| *ANXA5* | *CA9* | *CDSN* | *CYP3A4* | *ETHE1* | *GLI1* | *HTATIP2* | *KPNA2* | *MGAM* | *NR1H4* | *PNP* | *RHOA* | *SMAD4* | *TIMP1* | *YBX1* |
| *ANXA6* | *CABIN1* | *CDX2* | *CYP3A5* | *ETS1* | *GLI2* | *HTT* | *KRAS* | *MGAT5* | *NR1I2* | *PNPLA2* | *RHOC* | *SMAD7* | *TIMP2* | *YY1* |
| *ANXA7* | *CALB1* | *CEACAM1* | *CYP3A7* | *ETS2* | *GLP1R* | *ICAM1* | *KRT18* | *MGMT* | *NR1I3* | *PNPLA3* | *RIMS2* | *SMARCB1* | *TIMP3* | *YY1AP1* |
| *APAF1* | *CALB2* | *CEACAM5* | *CYP4F3* | *ETV6* | *GLS* | *ID1* | *KRT19* | *MIA3* | *NR2C2* | *POLB* | *RIPK1* | *SMS* | *TIPARP* | *ZBTB7A* |
| *APC* | *CALCA* | *CEBPA* | *CYP7A1* | *EZH2* | *GLUL* | *IDE* | *KRT20* | *MIB1* | *NR2F1* | *POLD1* | *RITA1* | *SMYD3* | *TJP1* | *ZEB1* |
| *APCS* | *CALM1* | *CEBPB* | *CYR61* | *EZR* | *GLYAT* | *IFNA1* | *KRT7* | *MIR17HG* | *NR2F2* | *POLDIP3* | *RNASE1* | *SNAI1* | *TKT* | *ZEB2* |
| *APEX1* | *CALM2* | *CENPJ* | *DAND5* | *F2* | *GMPS* | *IFNA2* | *KRT8* | *MKI67* | *NR3C1* | *POMC* | *ROCK1* | *SNCA* | *TLR2* | *ZGLP1* |
| *APOA1* | *CALM3* | *CES1* | *DDIT3* | *F7* | *GNMT* | *IFNAR2* | *LAMA2* | *MLANA* | *NR3C2* | *POU2F1* | *ROCK2* | *SND1* | *TLR3* | *ZHX2* |
| *APOA2* | *CALR* | *CETP* | *DDX19A* | *F8* | *GOLM1* | *IFNB1* | *LAMA4* | *MLH1* | *NR4A1* | *POU5F1* | *RPL17* | *SNRPE* | *TLR4* | *ZMYM2* |
| *APOA4* | *CANX* | *CFB* | *DDX20* | *FABP1* | *GOT2* | *IFNG* | *LAMC1* | *MME* | *NR5A2* | *PPA1* | *RPS6* | *SOAT1* | *TM4SF5* | *ZNF135* |
| *APOB* | *CAP2* | *CFLAR* | *DDX3X* | *FADD* | *GPC1* | *IFNL3* | *LAMTOR5* | *MMP1* | *NRAS* | *PPARA* | *RPS6KA3* | *SOCS1* | *TM6SF2* | *ZNF331* |
| *APOBEC1* | *CAPNS1* | *CFTR* | *DDX43* | *FAH* | *GPC3* | *IGF1* | *LAPTM4A* | *MMP13* | *NRSN2* | *PPARD* | *RPS6KB1* | *SOCS3* | *TMEM260* | *ZNF77* |
| *APOC3* | *CASP1* | *CHAT* | *DDX46* | *FAS* | *GPNMB* | *IGF1R* | *LAPTM4B* | *MMP14* | *NSG1* | *PPARG* | *RSF1* | *SOD2* | *TNF* | *ZSCAN1* |
| *APOE* | *CASP2* | *CHD1L* | *DDX58* | *FASLG* | *GPX1* | *IGF2* | *LARP6* | *MMP2* | *NT5C1B* | *PPARGC1A* | *RSRP1* | *SOX2* | *TNFRSF10A* | |
| *APP* | *CASP3* | *CHEK1* | *DECR1* | *FASN* | *GPX4* | *IGF2BP1* | *LATS1* | *MMP7* | *NT5E* | *PPAT* | *RTL10* | *SOX4* | *TNFRSF10B* | |
| *AQP7* | *CASP4* | *CHEK2* | *DEPDC5* | *FBN1* | *GRAP2* | *IGF2BP2* | *LDHA* | *MMP9* | *NTF3* | *PPIA* | *RUNX2* | *SP1* | *TNFRSF10C* | |
| *AQP9* | *CASP7* | *CHGA* | *DERL2* | *FBXW7* | *GRB2* | *IGF2BP3* | *LDLR* | *MPO* | *NUP62* | *PPL* | *RUNX3* | *SPHK1* | *TNFRSF1A* |  |
| *AR* | *CASP8* | *CHRNA4* | *DES* | *FDFT1* | *GRIN1* | *IGF2R* | *LEF1* | *MPV17* | *OAT* | *PPM1B* | *RXRA* | *SPP1* | *TNFRSF6B* |  |
| *ARCN1* | *CASP9* | *CHUK* | *DGUOK* | *FGF19* | *GRN* | *IGFBP1* | *LEP* | *MRI1* | *ODC1* | *PPOX* | *S100A4* | *SPPL2A* | *TNFRSF8* |  |
| *ARG1* | *CAT* | *CIB1* | *DHFR* | *FGF2* | *GRP* | *IGFBP3* | *LEPR* | *MRPS11* | *OGG1* | *PPP1R13L* | *S100A9* | *SQSTM1* | *TNFSF10* |  |
| *ARHGAP24* | *CAV1* | *CIP2A* | *DHX16* | *FGF21* | *GSK3B* | *IKBKG* | *LGALS1* | *MS4A1* | *ONECUT1* | *PPP1R1A* | *SAA1* | *SRC* | *TNFSF11* |  |
| *ARID1A* | *CBLL2* | *CISH* | *DIABLO* | *FGFR1* | *GSN* | *IL10* | *LGALS3* | *MSH3* | *OSM* | *PRDM2* | *SAA2* | *SRD5A2* | *TNRC6B* |  |
| *ARID2* | *CCK* | *CLDN1* | *DKK1* | *FGFR2* | *GSR* | *IL11* | *LGALS9* | *MSI2* | *OTC* | *PRDX3* | *SALL4* | *SREBF1* | *TNS1* |  |
| *ARNT* | *CCL14* | *CLDN7* | *DLC1* | *FGFR4* | *GSTA2* | *IL13* | *LGR5* | *MST1* | *P2RY12* | *PRDX5* | *SARNP* | *SREBF2* | *TOGARAM1* | |
| *ARTN* | *CCL15* | *CLMP* | *DLEC1* | *FHIT* | *GSTM1* | *IL17D* | *LIF* | *MT1G* | *PAH* | *PRF1* | *SARS* | *SRF* | *TP53* |  |


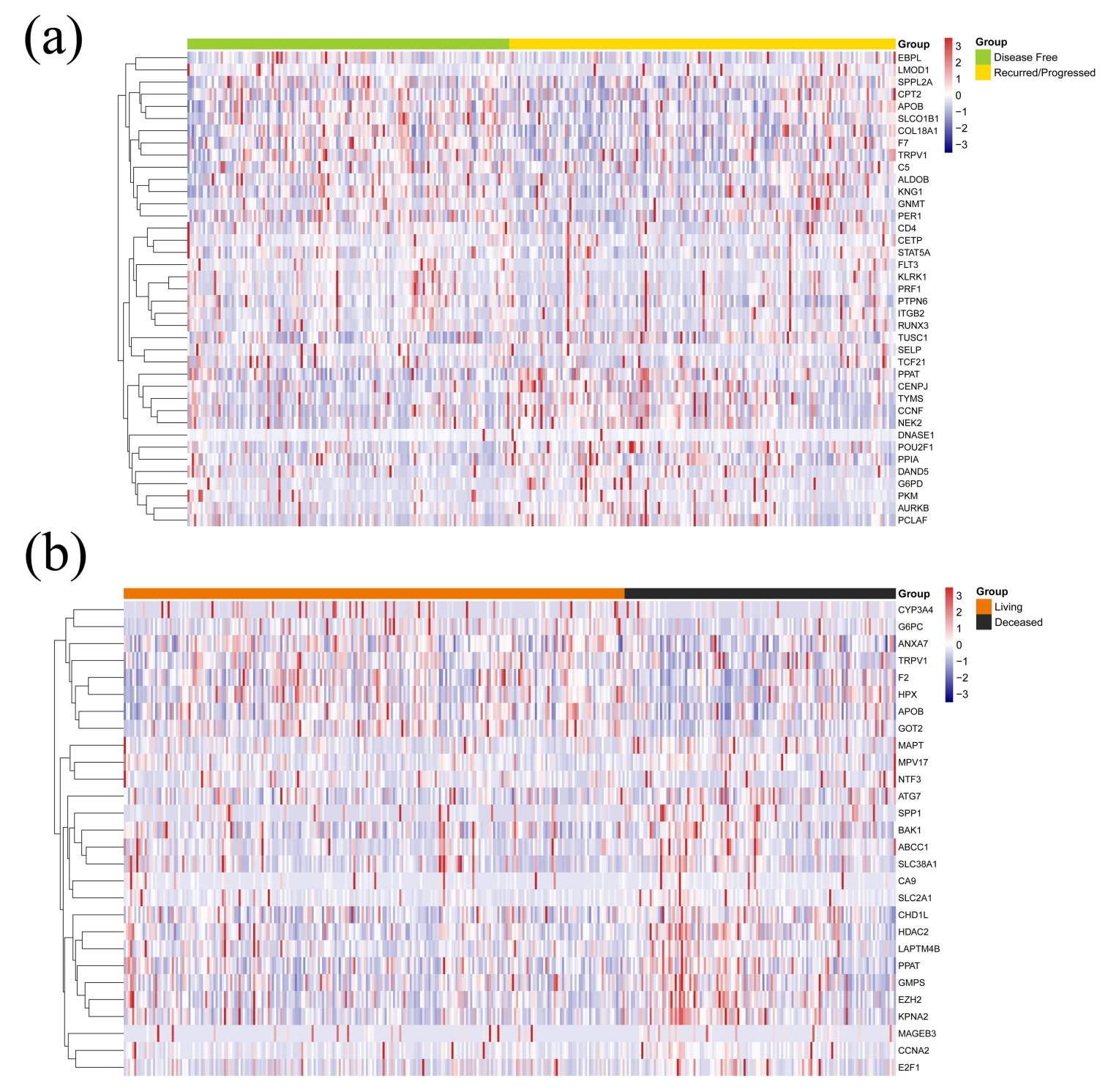
**Supplementary Figure S1.** Heat maps of the expression of 39 DFS-related genes and 28 OS-related genes in 1173 TCGA liver cancer samples

**Supplementary Figure S1.** Heat maps of the expression of 39 genes and 28 genes significantly associated with DFS and OS in 1173 TCGA liver cancer samples, respectively, which grouped by prognosis status. Heat maps were performed using the pheatmap function in R. **(A)** Heat map of the expression of 39 genes significantly associated with DFS in 1173 TCGA liver cancer samples, which grouped by prognosis status: disease free and recurred/progressed. **(B)** Heat map of the expression of 28 genes significantly associated with OS in 1173 TCGA liver cancer samples, which grouped by prognosis status: living and deceased.
